# Synthesis and crystal structure of a disubstituted nickel(II) bis­[(di­methyl­amino­phenyl­imino)­eth­yl]pyridine chloride complex

**DOI:** 10.1107/S2056989017010088

**Published:** 2017-07-13

**Authors:** Morgan Matthews, Madison Sendzik, Adrienne Bruggeman, Claire Kearns, Allen G. Oliver, Dominic C. Babbini

**Affiliations:** a Department of Chemistry and Physics, Saint Mary’s College, Notre Dame, IN 46556, USA; b Department of Chemistry and Biochemistry, University of Notre Dame, Notre Dame, IN 46556-5670, USA

**Keywords:** crystal structure, nickel complex, pyridine di­imine, redox non-innocent ligand, electron-donating groups

## Abstract

The solvated title compound, bis­[2,6-bis­(1-{[4-(di­methyl­amino)­phen­yl]imino-κ*N*}eth­yl)pyridine-κ*N*]nickel(II) dichloride–di­chloro­methane–water (1/2/2), [Ni(C_25_H_29_N_5_)_2_]Cl_2_·2CH_2_Cl_2_·2H_2_O, represents a nickel(II) bis­(pyridine di­imine) complex with electron-donating di­methyl­amino­phenyl substituents. The complex crystallizes as a water/di­chloro­methane solvate with *Z*′ = 2, thus the asymmetric unit consists of two Ni^II^ complex cations, four chloride anions, four adventitious water and four di­chloro­methane solvent mol­ecules. Around each octa­hedrally coordinated Ni^II^ cation, one pendant phenyl group on each of the two ligands has an intra­molecular π–π inter­action with the pyridine ring of the other chelating ligand. In the crystal, pairs of water mol­ecules are hydrogen bonded to pairs of chlorine atoms. The di­chloro­methane solvent mol­ecules are likewise hydrogen bonded to the chloride anions.

## Abstract 2

The synthesis and structural determination of a nickel(II) complex where the two nickel cations in the asymmetric unit each are coordinated by two tridentate, potentially redox non-innocent bis-imino­pyridyl ligands are reported.

## Chemical context   

Non-innocent ligand systems in organometallics can produce secondary reactivity and allow for unique mechanistic and redox properties (Babbini & Iluc, 2015 ▸; Praneeth *et al.*, 2012 ▸). Redox non-innocence is usually observed with chelate ligands which possess low-lying π-systems that can allow for electron transfer (Lyaskovskyy & de Bruin, 2012 ▸). These ligand systems can also allow for multiple-electron redox events to take place on metal cations which are usually relegated to single-electron events (Haneline & Heyduk, 2006 ▸). This can allow for the utilization of benign and economically viable base metal catalysts in lieu of traditional noble-metal catalysts (Chirik & Wieghardt, 2010 ▸). The development of new and varied organometallic complexes is essential for understanding the structure–property relationships, which give rise to redox non-innocence properties. With expanding inter­est in redox-active organometallic systems, we report here the synthesis and structural determination of a potentially redox-active nickel(II) complex possessing two pyridine di­imine ligands containing electron-donating substituents.

## Structural commentary   

**Figure 1:**
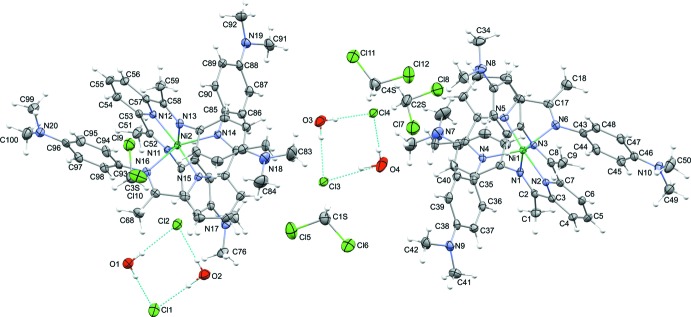
Selective labelling scheme for [Ni(C_25_H_29_N_5_)_2_]Cl_2_·2CH_2_Cl_2_·2H_2_O. Atomic displacement ellipsoids are depicted at the 50% probability level and H atoms shown as spheres of arbitrary radius. Hydrogen-bonding inter­actions are denoted as blue, dashed lines.

The title compounds crystallizes with two complex Ni^II^ cations, associated chloride anions, adventitious water and di­chloro­methane mol­ecules of solvation in the asymmetric unit (Fig. 1 ▸). Although the two cations are crystallographically independent they are chemically identical and the general discussion for one, holds for the second mol­ecule. We inspected the structure for higher and missed symmetry (Spek, 2009 ▸), but there is none.
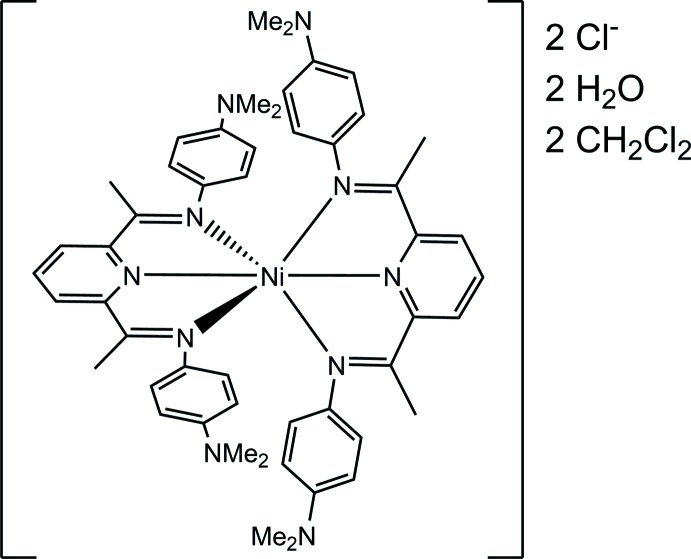


Each nickel(II) cation is coordinated in a distorted octa­hedral geometry by the imine and pyridine nitro­gen atoms of the two tridentate 2,6-bis­[1-(4-di­methyl­amino­phenyl­imino)­eth­yl]pyridine (PDI-DMA) ligands (Fig. 1 ▸, Table 1 ▸ for numerical details). The derived metrics for the mol­ecules are as expected. It should be noted that the Ni—N_py_ bond lengths are all considerably shorter than the Ni—N_imine_ bond lengths. However, both inter­actions are typical for these types of bonds/moieties.

**Figure 2:**
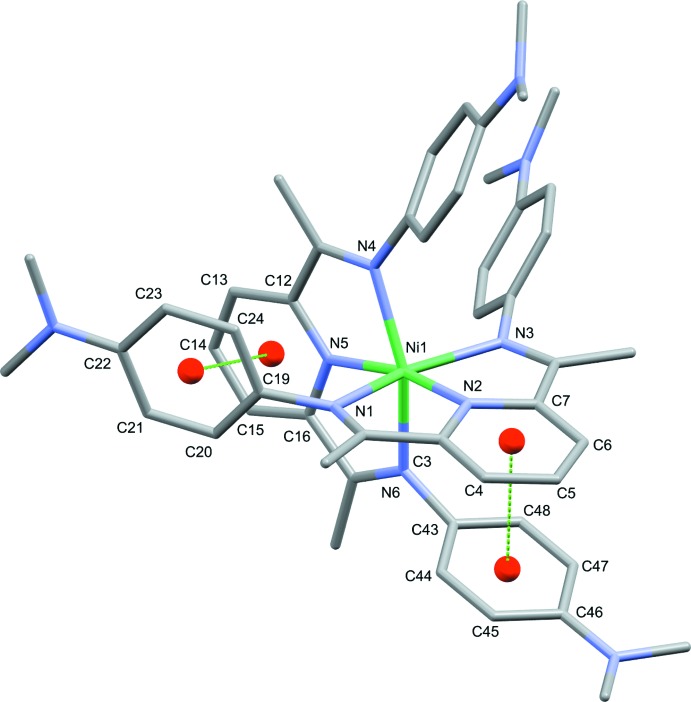
A single cationic unit (Ni1) displaying intra­molecular π–π inter­actions between phenyl and pyridyl rings.

An inter­esting feature of the cations is the orientation of the pendant di­methyl­amino­phenyl rings with respect to the pyridine ring of each ligand. In all cases, one pendant phenyl group is oriented close to perpendicular to the plane of the parent pyridine ring while the other is canted at an angle of around 60° (Fig. 2 ▸); numerical details of these features are collated in Table 2 ▸. Inspection of the mol­ecules shows that a combination of steric and π–π stacking inter­actions are the cause of these orientations. The phenyl rings that are close to perpendicular to the parent pyridine are sterically constrained by the pyridine rings of the second ligand and include weak intra­molecular π–π inter­actions (Table 2 ▸). The second di­methyl­amino­phenyl group is less hindered and adopts a typical tilted orientation.

## Supra­molecular features   

**Figure 3:**
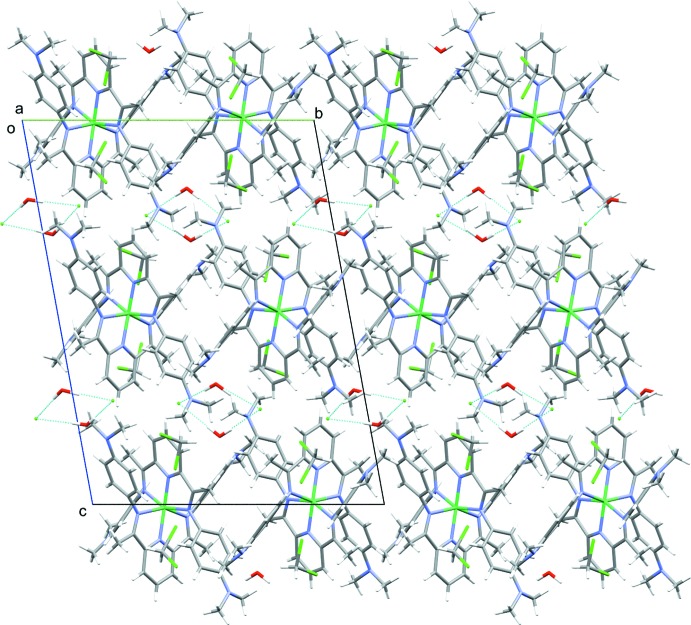
Packing diagram of [Ni(C_25_H_29_N_5_)_2_]Cl_2_·2CH_2_Cl_2_·2H_2_O, viewed along the *a* axis.

Within the inter­molecular packing of the cationic mol­ecules pairs of solvent water mol­ecules form a hydrogen-bonded dimer with pairs of chloride anions (Fig. 1 ▸, Table 3 ▸). Each di­chloro­methane solvent mol­ecule also forms a weak, but directional, hydrogen bond with a chloride anion (Table 3 ▸). Surprisingly, the cation does not have any particular directional inter­actions with other species in the structure, aside from a weak C—H⋯N inter­action with one of the di­chloro­methane solvents (Table 3 ▸) and typical van der Waals contacts (Fig. 3 ▸).

## Database survey   

A search of the Cambridge Structure Database (Version 5.38 + three updates; Groom *et al.*, 2016 ▸) reveals 13 structures that incorporate Ni^II^ coordinated by two bis-imino­aryl­pyridyl ligands. Of these, there are only three related structures with the imine carbon atoms methyl­ated (FADFUN, Patel *et al.*, 2010 ▸; MEGDUX, de Bruin *et al.*, 2000 ▸; QEZJOV, Trivedi *et al.*, 2007 ▸).

## Synthesis and crystallization   

The reagent 2,6-di­acetyl­pyridine was synthesized according to a previously reported method (Su & Feng, 2010 ▸). The ligand was prepared by a modification of previously reported Schiff-base condensation methods (Small & Brookhart, 1999 ▸; Chen *et al.*, 2003 ▸). All other reagents and solvents were purchased commercially and used without further purification. ^1^H NMR data were collected on a Varian 60 MHz NMR. Mass spectra were collected using direct injection on a ThermoScientific TSQ–ESI Mass spectrometer.

**Synthesis of 2,6-bis­(1-(4-di­methyl­amino­phenyl­imino)­eth­yl)pyridine (PDI-DMA).** A solution of 2,6-di­acetyl­pyridine (1.0 g, 6.10 mmol), 4-(di­methyl­amino)­aniline (1.7 g, 12.5 mmol) and formic acid (1 ml) was prepared in toluene (100 ml) under nitro­gen atmosphere and then stirred for 12 h on mol­ecular sieves. The reaction mixture was filtered and extracted with excess di­chloro­methane, then the amount of solvent was reduced *in vacuo*. The crude yellow product was then washed with cold methanol, followed by diethyl ether and filtered producing a pure bright-yellow solid (yield 1.8 g, 72.7% yield). ^1^H NMR (60 MHz, CDCl_3_, 293 K): δ 8.4–8.2 (*m*, 2H, Py-*H*), 7.9–7.8 (*m*, 1H, Py-*H*), 6.8 (*m*, 8H, Ar-*H*) 3.0 (*s*, 12H, N-C*H*_3_), 2.5 (*s*, 6H, C*H*_3_). MS (ESI): 400.4 *m*/*z* [C_25_H_29_N_5_]H^+^.

**Synthesis of [bis-(2,6-bis-(1-(4-di­methyl­amino­phenyl­imino)­eth­yl)pyridine)­nickel(II)] chloride.** A solution of the PDI-DMA ligand (300 mg, 0.75 mmol) and nickel(II) chloride (48.7 mg, 0.38 mmol) was prepared in THF (15 ml) under nitro­gen atmosphere, then stirred for 12 h. The solution was filtered and extracted with di­chloro­methane. The solvent was removed *in vacuo* yielding a dark reddish-brown solid (yield 220 mg, 67.5% yield). X-ray diffraction quality crystals were isolated as red–brown blocks by vapor diffusion of hexa­nes into a saturated solution of the product and di­chloro­methane. The complex was NMR silent (paramagnetic). MS (ESI): *m*/*z* 855.5 [C_50_H_57_N_10_Ni]^+^.

## Refinement   

Crystal data, data collection and structure refinement details are summarized in Table 4 ▸. H atoms bonded to carbon were placed in geometric positions with C—H = 0.95, 0.99 and 0.98 Å and *U*_iso_(*H*) = 1.2 ×, 1.2 × or 1.5 × *U*_eq_(*C*) for aromatic, methyl­ene and methyl H atoms, successively. Water H atoms were initially located from a difference Fourier map and included in their initially observed positions and allowed to ride with the position of the parent oxygen atom. Displacement parameters of the hydrogen atom were freely refined. Two reflections (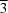
41 and 0,10,2) were omitted from the refinement for poorly agreeing statistics. It is not clear from the diffraction data why these two reflections agree poorly.

## Supplementary Material

Crystal structure: contains datablock(s) I. DOI: 10.1107/S2056989017010088/wm5402sup1.cif

Structure factors: contains datablock(s) I. DOI: 10.1107/S2056989017010088/wm5402Isup2.hkl

CCDC reference: 1560729

Additional supporting information:  crystallographic information; 3D view; checkCIF report

## Figures and Tables

**Table 1:** Selected geometric parameters (Å, °)

Ni1—N2	1.9694 (18)	Ni2—N12	1.9658 (18)
Ni1—N5	1.9725 (18)	Ni2—N15	1.9711 (18)
Ni1—N3	2.0844 (18)	Ni2—N14	2.0909 (19)
Ni1—N4	2.0948 (19)	Ni2—N11	2.0977 (19)
Ni1—N1	2.1243 (19)	Ni2—N13	2.1188 (19)
Ni1—N6	2.1354 (19)	Ni2—N16	2.141 (2)
			
N2—Ni1—N5	167.57 (8)	N12—Ni2—N15	167.94 (8)
N2—Ni1—N3	77.92 (7)	N12—Ni2—N14	110.67 (7)
N5—Ni1—N3	111.32 (7)	N15—Ni2—N14	78.01 (7)
N2—Ni1—N4	110.91 (7)	N12—Ni2—N11	77.79 (7)
N5—Ni1—N4	77.83 (8)	N15—Ni2—N11	111.03 (7)
N3—Ni1—N4	92.14 (7)	N14—Ni2—N11	91.70 (7)
N2—Ni1—N1	76.66 (7)	N12—Ni2—N13	76.89 (7)
N5—Ni1—N1	94.65 (7)	N15—Ni2—N13	94.86 (7)
N3—Ni1—N1	153.98 (7)	N14—Ni2—N13	91.88 (7)
N4—Ni1—N1	91.75 (7)	N11—Ni2—N13	154.04 (7)
N2—Ni1—N6	94.89 (7)	N12—Ni2—N16	94.74 (7)
N5—Ni1—N6	76.68 (8)	N15—Ni2—N16	76.75 (8)
N3—Ni1—N6	93.36 (7)	N14—Ni2—N16	154.58 (7)
N4—Ni1—N6	154.19 (7)	N11—Ni2—N16	94.50 (7)
N1—Ni1—N6	94.25 (7)	N13—Ni2—N16	93.21 (7)

**Table 2:** Inter­planar angles and close π–π inter­actions (Å, °)

Phenyl ring	Pyridine ring	Angle
C19–C24	N2–C7	87.58 (6)
C27–C28	N2–C7	64.85 (7)
C35–C40	N5–C16	54.42 (8)
C43–C48	N5–C16	83.76 (6)
C69–C74	N12–C57	63.47 (7)
C77–C82	N12–C57	87.83 (6)
C85–C90	N15–C66	53.63 (8)
C93–C98	N15–C66	81.53 (7)
		
Phenyl ring	Pyridine ring	*Cg*1⋯*Cg*2
C43–C48	N2–C7	3.487 (1)
C19–C24	N5–C46	3.675 (1)
C93–C98	N12–C57	3.520 (1)
C85–C90	N15–C66	3.696 (1)

*Cg*1 and *Cg*2 are the centroids for the named phenyl and pyridine rings.

**Table 3:** Hydrogen-bond geometry (Å, °)

*D*—H⋯*A*	*D*—H	H⋯*A*	*D*⋯*A*	*D*—H⋯*A*
O1—H1*OA*⋯Cl1	0.91	2.33	3.2381 (19)	177
O1—H1*OB*⋯Cl2	0.88	2.31	3.188 (2)	177
O2—H2*OA*⋯Cl1	1.01	2.18	3.192 (2)	175
O2—H2*OB*⋯Cl2	0.95	2.32	3.2691 (19)	177
O3—H3*OA*⋯Cl4	0.90	2.30	3.198 (2)	178
O3—H3*OB*⋯Cl3	0.87	2.34	3.201 (2)	174
O4—H4*OB*⋯Cl3	1.08	2.12	3.180 (2)	168
O4—H4*OA*⋯Cl4	0.94	2.25	3.192 (2)	177
C1*S*—H1*SB*⋯Cl3	0.99	2.47	3.422 (3)	162
C2*S*—H2*SA*⋯Cl4	0.99	2.81	3.743 (4)	158
C2*S*—H2*SB*⋯Cl1^i^	0.99	2.66	3.642 (3)	169
C3*S*—H3*SA*⋯N17^ii^	0.99	2.42	3.403 (4)	173
C3*S*—H3*SB*⋯Cl2	0.99	2.49	3.447 (3)	163
C4*S*—H4*SA*⋯Cl4	0.99	2.54	3.527 (3)	176

Symmetry codes: (i) 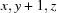; (ii) 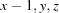.

**Table 4:** Experimental details

Crystal data	
Chemical formula	[Ni(C_25_H_29_N_5_)_2_]Cl_2_·2CH_2_Cl_2_·2H_2_O
*M*_r_	1134.55
Crystal system, space group	Triclinic, *P* 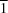
Temperature (K)	120
*a*, *b*, *c* (Å)	13.2227 (5), 17.7311 (6), 24.0242 (9)
α, β, γ (°)	79.6276 (12), 81.2551 (12), 89.3481 (12)
*V* (Å^3^)	5475.4 (3)
*Z*	4
Radiation type	Mo *K*α
μ (mm^−1^)	0.70
Crystal size (mm)	0.30 × 0.22 × 0.15

Data collection	
Diffractometer	Bruker Kappa X8 APEXII
Absorption correction	Numerical (*SADABS*; Krause *et al.*, 2015 ▸)
*T*_min_, *T*_max_	0.823, 0.917
No. of measured, independent and observed [*I* > 2σ(*I*)] reflections	69305, 24260, 17532
*R*_int_	0.034
(sin θ/λ)_max_ (Å^−1^)	0.643

Refinement	
*R*[*F* ^2^ > 2σ(*F* ^2^)], *wR*(*F* ^2^), *S*	0.045, 0.115, 1.04
No. of reflections	24260
No. of parameters	1315
H-atom treatment	H atoms treated by a mixture of independent and constrained refinement
Δρ_max_, Δρ_min_ (e Å^−3^)	1.19, −1.00

Computer programs: *APEX3* and *SAINT* (Bruker, 2016 ▸), *SHELXT* (Sheldrick, 2015*a* ▸), *SHELXL2014* (Sheldrick, 2015*b* ▸), *Mercury* (Macrae *et al.*, 2006 ▸), *POV-RAY* (Cason, 2003 ▸) and *publCIF* (Westrip, 2010 ▸).

## supplementary crystallographic information

### Crystal data

**Table:**

[Ni(C_25_H_29_N_5_)_2_]Cl_2_·2CH_2_Cl_2_·2H_2_O	*Z* = 4
*M**_r_* = 1134.55	*F*(000) = 2376
Triclinic, *P*1	*D*_x_ = 1.376 Mg m^−^^3^
*a* = 13.2227 (5) Å	Mo *K*α radiation, λ = 0.71073 Å
*b* = 17.7311 (6) Å	Cell parameters from 9951 reflections
*c* = 24.0242 (9) Å	θ = 2.3–27.1°
α = 79.6276 (12)°	µ = 0.70 mm^−^^1^
β = 81.2551 (12)°	*T* = 120 K
γ = 89.3481 (12)°	Block, red-brown
*V* = 5475.4 (3) Å^3^	0.30 × 0.22 × 0.15 mm

### Data collection

**Table:**

Bruker Kappa X8 APEXII diffractometer	24260 independent reflections
Radiation source: fine-focus sealed tube	17532 reflections with *I* > 2σ(*I*)
Graphite monochromator	*R*_int_ = 0.034
Detector resolution: 8.33 pixels mm^-1^	θ_max_ = 27.2°, θ_min_ = 0.9°
combination of ω and φ–scans	*h* = −16→17
Absorption correction: numerical (SADABS; Krause *et al.*, 2015)	*k* = −22→17
*T*_min_ = 0.823, *T*_max_ = 0.917	*l* = −30→30
69305 measured reflections	

### Refinement

**Table:**

Refinement on *F*^2^	Primary atom site location: dual
Least-squares matrix: full	Secondary atom site location: difference Fourier map
*R*[*F*^2^ > 2σ(*F*^2^)] = 0.045	Hydrogen site location: mixed
*wR*(*F*^2^) = 0.115	H atoms treated by a mixture of independent and constrained refinement
*S* = 1.04	*w* = 1/[σ^2^(*F*_o_^2^) + (0.0506*P*)^2^ + 2.6902*P*] where *P* = (*F*_o_^2^ + 2*F*_c_^2^)/3
24260 reflections	(Δ/σ)_max_ = 0.001
1315 parameters	Δρ_max_ = 1.19 e Å^−^^3^
0 restraints	Δρ_min_ = −1.00 e Å^−^^3^

### Special details

**Table:**

Geometry. All esds (except the esd in the dihedral angle between two l.s. planes) are estimated using the full covariance matrix. The cell esds are taken into account individually in the estimation of esds in distances, angles and torsion angles; correlations between esds in cell parameters are only used when they are defined by crystal symmetry. An approximate (isotropic) treatment of cell esds is used for estimating esds involving l.s. planes.

### Fractional atomic coordinates and isotropic or equivalent isotropic displacement parameters (Å^2^)

**Table:**

	*x*	*y*	*z*	*U*_iso_*/*U*_eq_	
Ni1	0.94318 (2)	0.75736 (2)	0.49692 (2)	0.01340 (7)	
N1	1.05673 (14)	0.67220 (11)	0.49125 (8)	0.0166 (4)	
N2	0.95646 (14)	0.71811 (10)	0.57750 (8)	0.0144 (4)	
N3	0.82707 (14)	0.81477 (10)	0.54041 (8)	0.0144 (4)	
N4	0.83769 (14)	0.69100 (11)	0.46895 (8)	0.0159 (4)	
N5	0.96256 (14)	0.79725 (11)	0.41383 (8)	0.0160 (4)	
N6	1.05128 (14)	0.84927 (11)	0.48658 (8)	0.0175 (4)	
N7	1.1429 (2)	0.59662 (16)	0.27296 (10)	0.0457 (7)	
N8	0.52711 (16)	0.97392 (12)	0.42701 (9)	0.0280 (5)	
N9	0.55000 (15)	0.49107 (12)	0.61881 (8)	0.0227 (5)	
N10	1.15686 (17)	0.90581 (13)	0.69285 (9)	0.0296 (5)	
C1	1.16227 (19)	0.57935 (14)	0.54579 (11)	0.0233 (5)	
H1A	1.136317	0.539445	0.578538	0.035*	
H1B	1.225916	0.601482	0.552529	0.035*	
H1C	1.175557	0.556835	0.511047	0.035*	
C2	1.08458 (17)	0.64073 (13)	0.53853 (10)	0.0168 (5)	
C3	1.03387 (17)	0.67150 (13)	0.58929 (10)	0.0167 (5)	
C4	1.06214 (18)	0.65800 (13)	0.64315 (10)	0.0194 (5)	
H4	1.116034	0.624052	0.651682	0.023*	
C5	1.00967 (19)	0.69538 (14)	0.68448 (10)	0.0228 (5)	
H5	1.028500	0.687638	0.721684	0.027*	
C6	0.93018 (18)	0.74383 (13)	0.67207 (9)	0.0194 (5)	
H6	0.893902	0.769471	0.700257	0.023*	
C7	0.90505 (17)	0.75385 (13)	0.61731 (9)	0.0161 (5)	
C8	0.82490 (17)	0.80492 (13)	0.59496 (9)	0.0173 (5)	
C9	0.7536 (2)	0.84156 (15)	0.63575 (10)	0.0260 (6)	
H9A	0.721265	0.885380	0.614604	0.039*	
H9B	0.791610	0.859229	0.663071	0.039*	
H9C	0.700707	0.804227	0.656481	0.039*	
C10	0.7904 (2)	0.65176 (15)	0.38197 (10)	0.0257 (6)	
H10A	0.841203	0.632636	0.353884	0.039*	
H10B	0.740456	0.682801	0.362100	0.039*	
H10C	0.755211	0.608311	0.408634	0.039*	
C11	0.84277 (17)	0.69971 (13)	0.41435 (9)	0.0168 (5)	
C12	0.91471 (18)	0.76121 (13)	0.38117 (9)	0.0186 (5)	
C13	0.9351 (2)	0.78081 (15)	0.32215 (10)	0.0248 (6)	
H13	0.900143	0.755688	0.298739	0.030*	
C14	1.0075 (2)	0.83771 (15)	0.29787 (10)	0.0289 (6)	
H14	1.021311	0.852980	0.257442	0.035*	
C15	1.05953 (19)	0.87230 (14)	0.33243 (10)	0.0238 (5)	
H15	1.110914	0.910294	0.316229	0.029*	
C16	1.03522 (18)	0.85042 (13)	0.39117 (10)	0.0185 (5)	
C17	1.08515 (17)	0.87935 (13)	0.43460 (10)	0.0184 (5)	
C18	1.17011 (19)	0.93686 (15)	0.41517 (11)	0.0261 (6)	
H18A	1.228120	0.913848	0.394099	0.039*	
H18B	1.191450	0.953266	0.448513	0.039*	
H18C	1.146690	0.981332	0.390108	0.039*	
C19	1.08983 (17)	0.64669 (13)	0.43826 (10)	0.0178 (5)	
C20	1.16176 (18)	0.68909 (14)	0.39652 (10)	0.0230 (5)	
H20	1.198077	0.730099	0.405527	0.028*	
C21	1.18083 (19)	0.67215 (15)	0.34221 (11)	0.0251 (6)	
H21	1.230742	0.701307	0.314221	0.030*	
C22	1.1274 (2)	0.61228 (15)	0.32767 (10)	0.0271 (6)	
C23	1.05641 (19)	0.56951 (14)	0.37042 (10)	0.0225 (5)	
H23	1.020520	0.527833	0.362044	0.027*	
C24	1.03783 (18)	0.58720 (13)	0.42482 (10)	0.0193 (5)	
H24	0.988637	0.557966	0.453214	0.023*	
C25	1.0827 (3)	0.53817 (19)	0.25846 (13)	0.0521 (9)	
H25A	1.094887	0.540549	0.216909	0.078*	
H25B	1.009999	0.546380	0.270993	0.078*	
H25C	1.102091	0.487698	0.277596	0.078*	
C26	1.2234 (3)	0.6350 (2)	0.23073 (12)	0.0498 (9)	
H26A	1.220798	0.619096	0.193961	0.075*	
H26B	1.289751	0.621567	0.242902	0.075*	
H26C	1.214523	0.690610	0.226508	0.075*	
C27	0.74965 (17)	0.85605 (13)	0.51336 (9)	0.0157 (5)	
C28	0.64669 (18)	0.83715 (13)	0.53030 (10)	0.0187 (5)	
H28	0.626768	0.797287	0.562145	0.022*	
C29	0.57299 (18)	0.87494 (14)	0.50192 (10)	0.0203 (5)	
H29	0.503067	0.860425	0.514036	0.024*	
C30	0.59985 (18)	0.93470 (13)	0.45527 (10)	0.0196 (5)	
C31	0.70425 (18)	0.95273 (14)	0.43811 (10)	0.0208 (5)	
H31	0.724853	0.992756	0.406481	0.025*	
C32	0.77730 (18)	0.91340 (13)	0.46633 (10)	0.0189 (5)	
H32	0.847651	0.925789	0.453346	0.023*	
C33	0.42161 (19)	0.94824 (15)	0.43961 (12)	0.0300 (6)	
H33A	0.381030	0.982107	0.414958	0.045*	
H33B	0.395252	0.949451	0.479827	0.045*	
H33C	0.416874	0.895724	0.432598	0.045*	
C34	0.5576 (2)	1.03030 (16)	0.37651 (12)	0.0341 (7)	
H34A	0.601731	1.006618	0.348058	0.051*	
H34B	0.595234	1.072150	0.386326	0.051*	
H34C	0.496755	1.050668	0.360655	0.051*	
C35	0.76478 (17)	0.63922 (13)	0.50586 (9)	0.0166 (5)	
C36	0.79552 (18)	0.58544 (13)	0.54891 (10)	0.0190 (5)	
H36	0.865580	0.583262	0.553655	0.023*	
C37	0.72591 (18)	0.53484 (14)	0.58508 (10)	0.0204 (5)	
H37	0.749218	0.497177	0.613513	0.024*	
C38	0.62107 (18)	0.53799 (14)	0.58066 (10)	0.0195 (5)	
C39	0.59132 (18)	0.59409 (14)	0.53729 (10)	0.0217 (5)	
H39	0.521086	0.597914	0.533023	0.026*	
C40	0.66142 (18)	0.64363 (14)	0.50083 (10)	0.0207 (5)	
H40	0.639019	0.681147	0.471984	0.025*	
C41	0.5864 (2)	0.42651 (15)	0.65642 (11)	0.0318 (6)	
H41A	0.621322	0.390774	0.633483	0.048*	
H41B	0.528145	0.400196	0.682571	0.048*	
H41C	0.634161	0.444938	0.678609	0.048*	
C42	0.4508 (2)	0.47877 (16)	0.60241 (11)	0.0310 (6)	
H42A	0.460600	0.459291	0.566317	0.047*	
H42B	0.414337	0.527378	0.597468	0.047*	
H42C	0.410640	0.441354	0.632386	0.047*	
C43	1.08716 (17)	0.87102 (13)	0.53470 (10)	0.0176 (5)	
C44	1.16933 (18)	0.83543 (14)	0.55673 (11)	0.0223 (5)	
H44	1.210560	0.802641	0.535813	0.027*	
C45	1.19257 (18)	0.84671 (14)	0.60863 (11)	0.0228 (5)	
H45	1.249412	0.821234	0.622969	0.027*	
C46	1.13421 (19)	0.89493 (14)	0.64076 (10)	0.0213 (5)	
C47	1.05252 (19)	0.93204 (14)	0.61710 (10)	0.0221 (5)	
H47	1.012341	0.966345	0.637060	0.026*	
C48	1.02924 (18)	0.91978 (13)	0.56543 (10)	0.0207 (5)	
H48	0.972696	0.945109	0.550648	0.025*	
C49	1.2292 (2)	0.85579 (16)	0.72049 (11)	0.0301 (6)	
H49A	1.297161	0.863652	0.697296	0.045*	
H49B	1.207223	0.802256	0.724323	0.045*	
H49C	1.232031	0.867663	0.758518	0.045*	
C50	1.0876 (3)	0.94759 (19)	0.72748 (12)	0.0452 (8)	
H50A	1.084052	1.000644	0.707531	0.068*	
H50B	1.111921	0.947165	0.764081	0.068*	
H50C	1.019373	0.923509	0.734519	0.068*	
Ni2	0.56947 (2)	0.24363 (2)	0.00835 (2)	0.01385 (7)	
N11	0.68705 (14)	0.18659 (10)	−0.03550 (8)	0.0155 (4)	
N12	0.55080 (14)	0.27763 (10)	−0.07190 (8)	0.0151 (4)	
N13	0.45280 (14)	0.32605 (11)	0.01389 (8)	0.0171 (4)	
N14	0.67462 (14)	0.31505 (11)	0.03144 (8)	0.0161 (4)	
N15	0.55654 (14)	0.20771 (11)	0.09172 (8)	0.0150 (4)	
N16	0.46245 (14)	0.14972 (11)	0.02362 (8)	0.0182 (4)	
N17	0.99169 (15)	0.04118 (12)	0.08291 (9)	0.0242 (5)	
N18	0.37685 (19)	0.41271 (14)	0.22925 (9)	0.0357 (6)	
N19	0.94578 (16)	0.51957 (12)	−0.12614 (8)	0.0237 (5)	
N20	0.31106 (17)	0.09062 (12)	−0.17089 (9)	0.0262 (5)	
C51	0.7577 (2)	0.15726 (15)	−0.13050 (10)	0.0269 (6)	
H51A	0.718818	0.137060	−0.156353	0.040*	
H51B	0.792933	0.115188	−0.109264	0.040*	
H51C	0.808292	0.195271	−0.152867	0.040*	
C52	0.68663 (17)	0.19410 (13)	−0.08951 (10)	0.0171 (5)	
C53	0.60277 (17)	0.24198 (13)	−0.11123 (9)	0.0166 (5)	
C54	0.57646 (19)	0.24980 (14)	−0.16545 (10)	0.0213 (5)	
H54	0.613553	0.224199	−0.193412	0.026*	
C55	0.49485 (19)	0.29578 (15)	−0.17801 (10)	0.0238 (5)	
H55	0.475471	0.302140	−0.215040	0.029*	
C56	0.44113 (18)	0.33270 (14)	−0.13672 (10)	0.0206 (5)	
H56	0.385370	0.364835	−0.145040	0.025*	
C57	0.47085 (17)	0.32147 (13)	−0.08330 (10)	0.0167 (5)	
C58	0.41979 (17)	0.35308 (13)	−0.03301 (10)	0.0166 (5)	
C59	0.33651 (19)	0.40976 (14)	−0.03978 (11)	0.0241 (6)	
H59A	0.358257	0.450045	−0.073032	0.036*	
H59B	0.321669	0.432684	−0.005289	0.036*	
H59C	0.274825	0.383835	−0.045475	0.036*	
C60	0.72346 (19)	0.36265 (15)	0.11472 (10)	0.0250 (6)	
H60A	0.779031	0.336004	0.132491	0.037*	
H60B	0.673451	0.379628	0.144231	0.037*	
H60C	0.751460	0.407272	0.086472	0.037*	
C61	0.67234 (17)	0.30942 (13)	0.08571 (9)	0.0172 (5)	
C62	0.60561 (18)	0.24702 (13)	0.12198 (10)	0.0185 (5)	
C63	0.59120 (19)	0.22916 (15)	0.18118 (10)	0.0236 (5)	
H63	0.626434	0.256967	0.202846	0.028*	
C64	0.5243 (2)	0.16992 (15)	0.20789 (10)	0.0275 (6)	
H64	0.514948	0.155467	0.248399	0.033*	
C65	0.47086 (19)	0.13146 (15)	0.17613 (10)	0.0242 (6)	
H65	0.423395	0.091485	0.194332	0.029*	
C66	0.48824 (17)	0.15267 (13)	0.11720 (10)	0.0179 (5)	
C67	0.43353 (17)	0.12126 (13)	0.07645 (10)	0.0176 (5)	
C68	0.34921 (18)	0.06396 (14)	0.09959 (10)	0.0237 (5)	
H68A	0.292197	0.088299	0.120406	0.036*	
H68B	0.374153	0.021270	0.125654	0.036*	
H68C	0.325786	0.044606	0.067920	0.036*	
C69	0.76642 (17)	0.14784 (13)	−0.00836 (9)	0.0157 (5)	
C70	0.86890 (18)	0.16776 (14)	−0.02611 (10)	0.0197 (5)	
H70	0.888006	0.206212	−0.059009	0.024*	
C71	0.94330 (18)	0.13256 (14)	0.00329 (10)	0.0198 (5)	
H71	1.013015	0.147165	−0.009612	0.024*	
C72	0.91799 (18)	0.07570 (13)	0.05183 (10)	0.0187 (5)	
C73	0.81430 (17)	0.05617 (13)	0.06959 (10)	0.0186 (5)	
H73	0.794696	0.017490	0.102280	0.022*	
C74	0.74035 (18)	0.09244 (13)	0.04017 (9)	0.0181 (5)	
H74	0.670321	0.079178	0.053433	0.022*	
C75	1.09837 (18)	0.04733 (15)	0.05675 (12)	0.0283 (6)	
H75A	1.107647	0.022214	0.023212	0.042*	
H75B	1.141015	0.022376	0.084491	0.042*	
H75C	1.118476	0.101550	0.044875	0.042*	
C76	0.9646 (2)	−0.02654 (15)	0.12591 (11)	0.0296 (6)	
H76A	0.904829	−0.016017	0.152782	0.044*	
H76B	1.022231	−0.040681	0.146707	0.044*	
H76C	0.948218	−0.068858	0.107401	0.044*	
C77	0.42147 (17)	0.35379 (13)	0.06615 (9)	0.0173 (5)	
C78	0.47223 (18)	0.41554 (14)	0.07687 (10)	0.0197 (5)	
H78	0.518429	0.445066	0.046939	0.024*	
C79	0.45709 (19)	0.43536 (14)	0.13060 (10)	0.0228 (5)	
H79	0.492865	0.478365	0.137058	0.027*	
C80	0.3896 (2)	0.39279 (15)	0.17561 (10)	0.0239 (5)	
C81	0.33736 (19)	0.33057 (15)	0.16367 (11)	0.0251 (6)	
H81	0.290104	0.301186	0.193112	0.030*	
C82	0.35333 (18)	0.31143 (14)	0.11014 (10)	0.0213 (5)	
H82	0.317470	0.268829	0.103109	0.026*	
C83	0.4592 (3)	0.4527 (2)	0.24455 (13)	0.0533 (9)	
H83A	0.523400	0.426066	0.235860	0.080*	
H83B	0.445762	0.454245	0.285587	0.080*	
H83C	0.464636	0.505161	0.222671	0.080*	
C84	0.3087 (3)	0.3664 (2)	0.27473 (12)	0.0478 (8)	
H84A	0.241338	0.363136	0.262939	0.072*	
H84B	0.301969	0.389675	0.309104	0.072*	
H84C	0.336403	0.314761	0.283087	0.072*	
C85	0.74349 (17)	0.36746 (13)	−0.00769 (10)	0.0172 (5)	
C86	0.84744 (18)	0.36710 (14)	−0.00498 (10)	0.0214 (5)	
H86	0.873301	0.331411	0.023720	0.026*	
C87	0.91370 (19)	0.41760 (14)	−0.04322 (10)	0.0225 (5)	
H87	0.984552	0.416525	−0.040252	0.027*	
C88	0.87861 (18)	0.47075 (14)	−0.08657 (10)	0.0196 (5)	
C89	0.77311 (18)	0.47017 (14)	−0.08890 (10)	0.0203 (5)	
H89	0.746454	0.506014	−0.117197	0.024*	
C90	0.70755 (18)	0.41854 (14)	−0.05086 (10)	0.0191 (5)	
H90	0.636926	0.417942	−0.054258	0.023*	
C91	1.04661 (19)	0.53340 (16)	−0.11291 (11)	0.0295 (6)	
H91A	1.085777	0.568007	−0.145205	0.044*	
H91B	1.082341	0.484664	−0.105975	0.044*	
H91C	1.039846	0.556860	−0.078608	0.044*	
C92	0.9038 (2)	0.58500 (16)	−0.16033 (11)	0.0312 (6)	
H92A	0.958874	0.612516	−0.188309	0.047*	
H92B	0.872168	0.619454	−0.135292	0.047*	
H92C	0.852204	0.567199	−0.180471	0.047*	
C93	0.41735 (17)	0.12778 (13)	−0.02151 (9)	0.0175 (5)	
C94	0.32708 (18)	0.15923 (14)	−0.03611 (11)	0.0237 (5)	
H94	0.288481	0.189838	−0.012175	0.028*	
C95	0.29206 (19)	0.14689 (14)	−0.08494 (11)	0.0257 (6)	
H95	0.229322	0.168900	−0.093819	0.031*	
C96	0.34662 (19)	0.10272 (13)	−0.12180 (10)	0.0208 (5)	
C97	0.43628 (19)	0.06970 (14)	−0.10535 (11)	0.0236 (5)	
H97	0.474148	0.037587	−0.128340	0.028*	
C98	0.47125 (18)	0.08247 (13)	−0.05667 (10)	0.0207 (5)	
H98	0.533245	0.059857	−0.047081	0.025*	
C99	0.2497 (2)	0.15044 (16)	−0.19891 (11)	0.0339 (7)	
H99A	0.228728	0.134516	−0.232516	0.051*	
H99B	0.290311	0.198064	−0.210936	0.051*	
H99C	0.188793	0.158934	−0.172099	0.051*	
C100	0.3762 (2)	0.05017 (18)	−0.20938 (12)	0.0392 (7)	
H10D	0.341524	0.044890	−0.241766	0.059*	
H10E	0.390724	−0.000810	−0.188909	0.059*	
H10F	0.440548	0.079042	−0.223531	0.059*	
Cl1	0.32592 (6)	−0.13439 (4)	0.27016 (3)	0.03678 (17)	
Cl2	0.20244 (5)	0.14063 (4)	0.22310 (3)	0.02772 (14)	
Cl3	0.78142 (5)	0.36539 (4)	0.24816 (3)	0.02773 (15)	
Cl4	0.68265 (5)	0.64160 (4)	0.26307 (3)	0.02594 (14)	
C1S	0.5820 (2)	0.32718 (17)	0.35855 (12)	0.0371 (7)	
H1SA	0.531364	0.368201	0.362327	0.045*	
H1SB	0.641193	0.348995	0.330020	0.045*	
Cl5	0.52651 (6)	0.25141 (5)	0.33470 (4)	0.0515 (2)	
Cl6	0.62287 (6)	0.29465 (5)	0.42512 (3)	0.04281 (19)	
C2S	0.5202 (3)	0.76821 (19)	0.34787 (13)	0.0475 (9)	
H2SA	0.545927	0.724886	0.329145	0.057*	
H2SB	0.475215	0.799167	0.323530	0.057*	
Cl7	0.44865 (7)	0.73200 (5)	0.41754 (4)	0.0514 (2)	
Cl8	0.62339 (7)	0.82496 (5)	0.35527 (3)	0.0502 (2)	
C3S	0.0469 (2)	0.20879 (16)	0.12292 (12)	0.0352 (7)	
H3SA	0.025519	0.159576	0.113933	0.042*	
H3SB	0.101700	0.197948	0.146971	0.042*	
Cl9	0.09611 (6)	0.27075 (4)	0.05845 (3)	0.04107 (18)	
Cl10	−0.05734 (6)	0.24757 (5)	0.16151 (4)	0.0514 (2)	
C4S	0.9087 (2)	0.65869 (17)	0.16231 (12)	0.0381 (7)	
H4SA	0.844761	0.651571	0.190230	0.046*	
H4SB	0.941460	0.608152	0.162716	0.046*	
Cl11	0.87950 (6)	0.69424 (4)	0.09336 (3)	0.04264 (19)	
Cl12	0.99139 (6)	0.72231 (5)	0.18242 (4)	0.0502 (2)	
O1	0.15979 (14)	−0.03002 (11)	0.20668 (7)	0.0298 (4)	
H1OA	0.208421	−0.057922	0.223474	0.049 (10)*	
H1OB	0.173321	0.016378	0.211974	0.061 (11)*	
O2	0.35472 (14)	0.03293 (11)	0.29766 (7)	0.0331 (4)	
H2OA	0.349578	−0.019509	0.287110	0.074 (12)*	
H2OB	0.310178	0.065391	0.277110	0.082 (13)*	
O3	0.70769 (17)	0.52151 (11)	0.17747 (8)	0.0409 (5)	
H3OA	0.702136	0.555309	0.201415	0.068 (12)*	
H3OB	0.732136	0.479689	0.194895	0.087 (14)*	
O4	0.8157 (2)	0.50164 (12)	0.31347 (10)	0.0597 (7)	
H4OA	0.774772	0.541506	0.298367	0.067 (11)*	
H4OB	0.799392	0.461176	0.287497	0.16 (2)*	

### Atomic displacement parameters (Å^2^)

**Table:**

	*U*^11^	*U*^22^	*U*^33^	*U*^12^	*U*^13^	*U*^23^
Ni1	0.01361 (15)	0.01447 (15)	0.01276 (14)	0.00145 (12)	−0.00309 (11)	−0.00332 (11)
N1	0.0149 (10)	0.0183 (10)	0.0183 (10)	0.0020 (8)	−0.0039 (8)	−0.0069 (8)
N2	0.0146 (10)	0.0134 (10)	0.0162 (9)	−0.0004 (8)	−0.0040 (7)	−0.0042 (8)
N3	0.0144 (10)	0.0126 (10)	0.0163 (10)	0.0001 (8)	−0.0020 (7)	−0.0027 (8)
N4	0.0154 (10)	0.0153 (10)	0.0189 (10)	0.0027 (8)	−0.0056 (8)	−0.0061 (8)
N5	0.0150 (10)	0.0156 (10)	0.0171 (10)	0.0045 (8)	−0.0023 (8)	−0.0027 (8)
N6	0.0136 (10)	0.0166 (10)	0.0228 (10)	0.0009 (8)	−0.0020 (8)	−0.0053 (8)
N7	0.0616 (18)	0.0553 (18)	0.0196 (12)	−0.0070 (14)	0.0054 (11)	−0.0146 (12)
N8	0.0188 (11)	0.0269 (12)	0.0361 (13)	0.0039 (9)	−0.0085 (9)	0.0036 (10)
N9	0.0185 (11)	0.0253 (12)	0.0244 (11)	−0.0042 (9)	−0.0017 (8)	−0.0054 (9)
N10	0.0341 (13)	0.0290 (13)	0.0323 (12)	0.0119 (10)	−0.0155 (10)	−0.0152 (10)
C1	0.0219 (13)	0.0217 (13)	0.0276 (13)	0.0070 (11)	−0.0080 (10)	−0.0044 (11)
C2	0.0134 (11)	0.0156 (12)	0.0227 (12)	−0.0015 (9)	−0.0051 (9)	−0.0049 (9)
C3	0.0165 (12)	0.0135 (12)	0.0210 (12)	−0.0017 (9)	−0.0066 (9)	−0.0021 (9)
C4	0.0214 (13)	0.0173 (12)	0.0200 (12)	0.0027 (10)	−0.0083 (10)	−0.0007 (10)
C5	0.0276 (14)	0.0259 (14)	0.0152 (12)	−0.0025 (11)	−0.0075 (10)	−0.0005 (10)
C6	0.0241 (13)	0.0195 (13)	0.0148 (11)	−0.0025 (10)	−0.0023 (9)	−0.0048 (9)
C7	0.0173 (12)	0.0150 (12)	0.0158 (11)	−0.0027 (9)	−0.0032 (9)	−0.0018 (9)
C8	0.0171 (12)	0.0168 (12)	0.0190 (12)	0.0001 (10)	−0.0022 (9)	−0.0060 (9)
C9	0.0288 (14)	0.0313 (15)	0.0197 (12)	0.0084 (12)	−0.0045 (10)	−0.0089 (11)
C10	0.0263 (14)	0.0323 (15)	0.0231 (13)	0.0001 (12)	−0.0089 (11)	−0.0130 (11)
C11	0.0162 (12)	0.0177 (12)	0.0185 (12)	0.0055 (10)	−0.0061 (9)	−0.0059 (9)
C12	0.0215 (13)	0.0194 (12)	0.0170 (11)	0.0075 (10)	−0.0059 (9)	−0.0069 (10)
C13	0.0325 (15)	0.0266 (14)	0.0172 (12)	0.0061 (12)	−0.0072 (10)	−0.0065 (10)
C14	0.0403 (16)	0.0309 (15)	0.0133 (12)	0.0062 (13)	0.0000 (11)	−0.0016 (11)
C15	0.0259 (14)	0.0217 (13)	0.0201 (12)	0.0034 (11)	0.0039 (10)	−0.0004 (10)
C16	0.0173 (12)	0.0169 (12)	0.0196 (12)	0.0059 (10)	0.0016 (9)	−0.0023 (9)
C17	0.0134 (12)	0.0149 (12)	0.0251 (13)	0.0037 (9)	0.0001 (9)	−0.0020 (10)
C18	0.0179 (13)	0.0268 (14)	0.0315 (14)	−0.0017 (11)	0.0002 (11)	−0.0029 (11)
C19	0.0167 (12)	0.0189 (12)	0.0187 (12)	0.0073 (10)	−0.0039 (9)	−0.0052 (10)
C20	0.0160 (12)	0.0214 (13)	0.0315 (14)	0.0038 (10)	−0.0018 (10)	−0.0063 (11)
C21	0.0204 (13)	0.0248 (14)	0.0258 (13)	0.0041 (11)	0.0043 (10)	−0.0001 (11)
C22	0.0315 (15)	0.0288 (15)	0.0203 (13)	0.0084 (12)	0.0010 (11)	−0.0069 (11)
C23	0.0267 (14)	0.0200 (13)	0.0228 (13)	0.0012 (11)	−0.0042 (10)	−0.0089 (10)
C24	0.0205 (13)	0.0160 (12)	0.0208 (12)	0.0026 (10)	−0.0020 (10)	−0.0024 (10)
C25	0.089 (3)	0.045 (2)	0.0248 (16)	0.0015 (19)	−0.0071 (16)	−0.0143 (14)
C26	0.051 (2)	0.069 (2)	0.0218 (15)	0.0097 (18)	0.0086 (14)	−0.0023 (15)
C27	0.0177 (12)	0.0152 (12)	0.0159 (11)	0.0033 (9)	−0.0041 (9)	−0.0061 (9)
C28	0.0187 (12)	0.0181 (12)	0.0184 (12)	−0.0005 (10)	−0.0003 (9)	−0.0027 (9)
C29	0.0137 (12)	0.0223 (13)	0.0257 (13)	−0.0003 (10)	−0.0014 (10)	−0.0075 (10)
C30	0.0166 (12)	0.0178 (12)	0.0251 (13)	0.0025 (10)	−0.0044 (10)	−0.0052 (10)
C31	0.0194 (13)	0.0185 (13)	0.0228 (12)	0.0002 (10)	−0.0034 (10)	0.0006 (10)
C32	0.0141 (12)	0.0184 (12)	0.0227 (12)	−0.0012 (10)	0.0002 (9)	−0.0024 (10)
C33	0.0181 (13)	0.0281 (15)	0.0461 (17)	0.0025 (11)	−0.0130 (12)	−0.0063 (12)
C34	0.0282 (15)	0.0335 (16)	0.0377 (16)	0.0062 (13)	−0.0125 (12)	0.0069 (13)
C35	0.0160 (12)	0.0174 (12)	0.0180 (11)	0.0004 (9)	−0.0039 (9)	−0.0067 (9)
C36	0.0150 (12)	0.0209 (13)	0.0233 (12)	0.0023 (10)	−0.0066 (10)	−0.0071 (10)
C37	0.0216 (13)	0.0198 (13)	0.0200 (12)	0.0014 (10)	−0.0044 (10)	−0.0035 (10)
C38	0.0183 (12)	0.0208 (13)	0.0218 (12)	0.0003 (10)	−0.0021 (10)	−0.0110 (10)
C39	0.0142 (12)	0.0293 (14)	0.0235 (13)	0.0013 (10)	−0.0040 (10)	−0.0092 (11)
C40	0.0204 (13)	0.0209 (13)	0.0227 (12)	0.0033 (10)	−0.0072 (10)	−0.0059 (10)
C41	0.0326 (16)	0.0252 (15)	0.0342 (15)	−0.0025 (12)	−0.0014 (12)	0.0004 (12)
C42	0.0250 (14)	0.0355 (16)	0.0325 (15)	−0.0094 (12)	−0.0036 (11)	−0.0063 (12)
C43	0.0146 (12)	0.0171 (12)	0.0212 (12)	−0.0031 (10)	−0.0024 (9)	−0.0038 (9)
C44	0.0176 (13)	0.0209 (13)	0.0311 (14)	0.0012 (10)	−0.0041 (10)	−0.0115 (11)
C45	0.0165 (12)	0.0219 (13)	0.0325 (14)	0.0037 (10)	−0.0083 (10)	−0.0081 (11)
C46	0.0214 (13)	0.0195 (13)	0.0249 (13)	−0.0016 (10)	−0.0063 (10)	−0.0069 (10)
C47	0.0224 (13)	0.0183 (13)	0.0272 (13)	0.0029 (10)	−0.0043 (10)	−0.0083 (10)
C48	0.0181 (12)	0.0177 (12)	0.0274 (13)	0.0008 (10)	−0.0061 (10)	−0.0046 (10)
C49	0.0318 (15)	0.0338 (16)	0.0254 (14)	0.0011 (12)	−0.0087 (11)	−0.0034 (12)
C50	0.057 (2)	0.055 (2)	0.0333 (16)	0.0227 (17)	−0.0205 (15)	−0.0239 (15)
Ni2	0.01312 (15)	0.01556 (15)	0.01360 (14)	0.00094 (12)	−0.00360 (11)	−0.00327 (11)
N11	0.0140 (10)	0.0143 (10)	0.0187 (10)	−0.0005 (8)	−0.0050 (8)	−0.0027 (8)
N12	0.0142 (10)	0.0142 (10)	0.0172 (10)	0.0002 (8)	−0.0041 (8)	−0.0024 (8)
N13	0.0147 (10)	0.0192 (10)	0.0187 (10)	0.0014 (8)	−0.0032 (8)	−0.0068 (8)
N14	0.0130 (10)	0.0167 (10)	0.0196 (10)	0.0016 (8)	−0.0053 (8)	−0.0035 (8)
N15	0.0124 (10)	0.0175 (10)	0.0159 (9)	0.0040 (8)	−0.0032 (7)	−0.0044 (8)
N16	0.0159 (10)	0.0173 (10)	0.0222 (10)	0.0014 (8)	−0.0026 (8)	−0.0061 (8)
N17	0.0173 (11)	0.0253 (12)	0.0309 (12)	0.0039 (9)	−0.0101 (9)	−0.0022 (9)
N18	0.0465 (15)	0.0418 (15)	0.0183 (11)	−0.0026 (12)	0.0030 (10)	−0.0106 (10)
N19	0.0212 (11)	0.0263 (12)	0.0224 (11)	−0.0051 (9)	0.0002 (9)	−0.0041 (9)
N20	0.0332 (13)	0.0223 (12)	0.0254 (11)	0.0000 (10)	−0.0118 (9)	−0.0038 (9)
C51	0.0314 (15)	0.0311 (15)	0.0215 (13)	0.0138 (12)	−0.0076 (11)	−0.0112 (11)
C52	0.0172 (12)	0.0156 (12)	0.0194 (12)	0.0002 (10)	−0.0040 (9)	−0.0046 (9)
C53	0.0178 (12)	0.0147 (12)	0.0181 (11)	−0.0019 (9)	−0.0048 (9)	−0.0033 (9)
C54	0.0244 (13)	0.0229 (13)	0.0171 (12)	0.0028 (11)	−0.0044 (10)	−0.0039 (10)
C55	0.0278 (14)	0.0286 (14)	0.0163 (12)	0.0024 (11)	−0.0099 (10)	−0.0023 (10)
C56	0.0199 (13)	0.0195 (13)	0.0236 (13)	0.0042 (10)	−0.0094 (10)	−0.0022 (10)
C57	0.0146 (12)	0.0144 (12)	0.0218 (12)	0.0002 (9)	−0.0051 (9)	−0.0030 (9)
C58	0.0132 (11)	0.0157 (12)	0.0221 (12)	−0.0005 (9)	−0.0055 (9)	−0.0038 (9)
C59	0.0224 (13)	0.0250 (14)	0.0263 (13)	0.0071 (11)	−0.0072 (10)	−0.0057 (11)
C60	0.0246 (14)	0.0296 (14)	0.0243 (13)	−0.0021 (11)	−0.0082 (10)	−0.0105 (11)
C61	0.0133 (12)	0.0210 (13)	0.0196 (12)	0.0026 (10)	−0.0077 (9)	−0.0053 (10)
C62	0.0175 (12)	0.0194 (12)	0.0199 (12)	0.0052 (10)	−0.0061 (9)	−0.0044 (10)
C63	0.0255 (14)	0.0281 (14)	0.0187 (12)	0.0050 (11)	−0.0085 (10)	−0.0043 (10)
C64	0.0323 (15)	0.0339 (15)	0.0150 (12)	0.0057 (12)	−0.0032 (11)	−0.0017 (11)
C65	0.0219 (13)	0.0254 (14)	0.0219 (13)	0.0015 (11)	0.0013 (10)	0.0007 (10)
C66	0.0149 (12)	0.0174 (12)	0.0209 (12)	0.0051 (10)	−0.0014 (9)	−0.0036 (10)
C67	0.0125 (11)	0.0160 (12)	0.0235 (12)	0.0023 (9)	−0.0007 (9)	−0.0036 (10)
C68	0.0175 (13)	0.0248 (14)	0.0265 (13)	−0.0025 (11)	0.0027 (10)	−0.0036 (11)
C69	0.0157 (12)	0.0174 (12)	0.0155 (11)	0.0028 (9)	−0.0042 (9)	−0.0059 (9)
C70	0.0191 (12)	0.0196 (13)	0.0199 (12)	−0.0006 (10)	−0.0031 (10)	−0.0015 (10)
C71	0.0128 (12)	0.0221 (13)	0.0246 (13)	0.0006 (10)	−0.0006 (9)	−0.0064 (10)
C72	0.0186 (12)	0.0182 (12)	0.0217 (12)	0.0012 (10)	−0.0059 (10)	−0.0080 (10)
C73	0.0169 (12)	0.0183 (12)	0.0199 (12)	−0.0001 (10)	−0.0032 (9)	−0.0012 (10)
C74	0.0147 (12)	0.0197 (12)	0.0196 (12)	−0.0004 (10)	−0.0016 (9)	−0.0036 (10)
C75	0.0180 (13)	0.0260 (14)	0.0443 (16)	0.0040 (11)	−0.0097 (12)	−0.0114 (12)
C76	0.0277 (15)	0.0309 (15)	0.0306 (14)	0.0078 (12)	−0.0118 (11)	−0.0010 (12)
C77	0.0144 (12)	0.0205 (13)	0.0183 (11)	0.0063 (10)	−0.0051 (9)	−0.0057 (10)
C78	0.0187 (12)	0.0189 (13)	0.0205 (12)	0.0023 (10)	−0.0018 (10)	−0.0023 (10)
C79	0.0259 (14)	0.0205 (13)	0.0236 (13)	0.0008 (11)	−0.0045 (10)	−0.0080 (10)
C80	0.0276 (14)	0.0248 (14)	0.0192 (12)	0.0072 (11)	−0.0022 (10)	−0.0057 (10)
C81	0.0210 (13)	0.0257 (14)	0.0250 (13)	0.0022 (11)	0.0047 (10)	−0.0019 (11)
C82	0.0142 (12)	0.0212 (13)	0.0294 (13)	0.0007 (10)	−0.0027 (10)	−0.0074 (10)
C83	0.075 (3)	0.063 (2)	0.0260 (16)	−0.014 (2)	−0.0068 (16)	−0.0183 (15)
C84	0.061 (2)	0.055 (2)	0.0230 (15)	0.0020 (18)	0.0058 (14)	−0.0063 (14)
C85	0.0156 (12)	0.0180 (12)	0.0193 (12)	−0.0011 (10)	−0.0029 (9)	−0.0061 (9)
C86	0.0200 (13)	0.0224 (13)	0.0229 (12)	0.0021 (10)	−0.0066 (10)	−0.0040 (10)
C87	0.0156 (12)	0.0271 (14)	0.0255 (13)	−0.0014 (10)	−0.0031 (10)	−0.0066 (11)
C88	0.0205 (13)	0.0217 (13)	0.0182 (12)	−0.0034 (10)	0.0006 (9)	−0.0104 (10)
C89	0.0216 (13)	0.0206 (13)	0.0192 (12)	−0.0001 (10)	−0.0054 (10)	−0.0029 (10)
C90	0.0145 (12)	0.0232 (13)	0.0218 (12)	0.0004 (10)	−0.0064 (9)	−0.0069 (10)
C91	0.0217 (14)	0.0330 (16)	0.0327 (15)	−0.0094 (12)	0.0023 (11)	−0.0077 (12)
C92	0.0334 (16)	0.0322 (16)	0.0245 (14)	−0.0057 (13)	0.0006 (11)	0.0001 (11)
C93	0.0154 (12)	0.0170 (12)	0.0196 (12)	−0.0051 (10)	−0.0014 (9)	−0.0029 (9)
C94	0.0178 (13)	0.0235 (14)	0.0324 (14)	0.0021 (10)	−0.0033 (10)	−0.0128 (11)
C95	0.0180 (13)	0.0249 (14)	0.0369 (15)	0.0049 (11)	−0.0104 (11)	−0.0077 (11)
C96	0.0221 (13)	0.0166 (12)	0.0230 (12)	−0.0057 (10)	−0.0046 (10)	−0.0009 (10)
C97	0.0247 (14)	0.0196 (13)	0.0280 (13)	0.0013 (11)	−0.0025 (11)	−0.0093 (10)
C98	0.0176 (12)	0.0185 (13)	0.0280 (13)	0.0036 (10)	−0.0052 (10)	−0.0078 (10)
C99	0.0399 (17)	0.0326 (16)	0.0302 (15)	0.0022 (13)	−0.0150 (13)	0.0001 (12)
C100	0.0505 (19)	0.0433 (18)	0.0288 (15)	0.0067 (15)	−0.0130 (13)	−0.0142 (13)
Cl1	0.0372 (4)	0.0388 (4)	0.0420 (4)	0.0156 (3)	−0.0163 (3)	−0.0197 (3)
Cl2	0.0301 (3)	0.0307 (4)	0.0248 (3)	0.0085 (3)	−0.0075 (3)	−0.0091 (3)
Cl3	0.0377 (4)	0.0244 (3)	0.0230 (3)	0.0058 (3)	−0.0118 (3)	−0.0037 (3)
Cl4	0.0318 (3)	0.0256 (3)	0.0227 (3)	0.0056 (3)	−0.0119 (3)	−0.0043 (2)
C1S	0.0398 (17)	0.0360 (17)	0.0377 (16)	0.0043 (14)	−0.0036 (13)	−0.0143 (13)
Cl5	0.0444 (5)	0.0626 (6)	0.0497 (5)	−0.0157 (4)	0.0080 (4)	−0.0278 (4)
Cl6	0.0417 (4)	0.0436 (4)	0.0466 (4)	0.0194 (4)	−0.0126 (3)	−0.0136 (4)
C2S	0.065 (2)	0.0424 (19)	0.0442 (18)	0.0049 (17)	−0.0361 (17)	−0.0097 (15)
Cl7	0.0575 (5)	0.0495 (5)	0.0516 (5)	0.0051 (4)	−0.0162 (4)	−0.0146 (4)
Cl8	0.0694 (6)	0.0399 (5)	0.0447 (5)	0.0032 (4)	−0.0117 (4)	−0.0145 (4)
C3S	0.0442 (18)	0.0299 (16)	0.0341 (15)	0.0086 (13)	−0.0093 (13)	−0.0107 (12)
Cl9	0.0470 (5)	0.0439 (4)	0.0336 (4)	−0.0046 (4)	−0.0058 (3)	−0.0104 (3)
Cl10	0.0378 (4)	0.0386 (5)	0.0687 (6)	0.0053 (4)	0.0097 (4)	−0.0011 (4)
C4S	0.0419 (18)	0.0387 (18)	0.0351 (16)	−0.0035 (14)	−0.0025 (13)	−0.0130 (13)
Cl11	0.0483 (5)	0.0393 (4)	0.0457 (4)	0.0161 (4)	−0.0162 (4)	−0.0152 (3)
Cl12	0.0410 (5)	0.0649 (6)	0.0500 (5)	−0.0092 (4)	−0.0063 (4)	−0.0249 (4)
O1	0.0311 (11)	0.0299 (11)	0.0288 (10)	0.0007 (9)	−0.0081 (8)	−0.0037 (8)
O2	0.0390 (12)	0.0301 (11)	0.0309 (10)	0.0060 (9)	−0.0106 (9)	−0.0035 (8)
O3	0.0683 (15)	0.0297 (11)	0.0323 (11)	0.0198 (11)	−0.0264 (10)	−0.0110 (9)
O4	0.0944 (19)	0.0404 (13)	0.0651 (15)	0.0388 (13)	−0.0599 (15)	−0.0272 (12)

### Geometric parameters (Å, º)

**Table:**

Ni1—N2	1.9694 (18)	N14—C61	1.285 (3)
Ni1—N5	1.9725 (18)	N14—C85	1.422 (3)
Ni1—N3	2.0844 (18)	N15—C62	1.331 (3)
Ni1—N4	2.0948 (19)	N15—C66	1.334 (3)
Ni1—N1	2.1243 (19)	N16—C67	1.280 (3)
Ni1—N6	2.1354 (19)	N16—C93	1.427 (3)
N1—C2	1.277 (3)	N17—C72	1.388 (3)
N1—C19	1.432 (3)	N17—C76	1.445 (3)
N2—C7	1.338 (3)	N17—C75	1.451 (3)
N2—C3	1.340 (3)	N18—C80	1.382 (3)
N3—C8	1.286 (3)	N18—C83	1.434 (4)
N3—C27	1.421 (3)	N18—C84	1.441 (4)
N4—C11	1.284 (3)	N19—C88	1.381 (3)
N4—C35	1.423 (3)	N19—C91	1.450 (3)
N5—C12	1.328 (3)	N19—C92	1.452 (3)
N5—C16	1.336 (3)	N20—C96	1.386 (3)
N6—C17	1.281 (3)	N20—C100	1.447 (3)
N6—C43	1.429 (3)	N20—C99	1.458 (3)
N7—C22	1.376 (3)	C51—C52	1.489 (3)
N7—C25	1.436 (4)	C51—H51A	0.9800
N7—C26	1.437 (4)	C51—H51B	0.9800
N8—C30	1.372 (3)	C51—H51C	0.9800
N8—C34	1.434 (3)	C52—C53	1.489 (3)
N8—C33	1.443 (3)	C53—C54	1.382 (3)
N9—C38	1.377 (3)	C54—C55	1.382 (3)
N9—C41	1.451 (3)	C54—H54	0.9500
N9—C42	1.456 (3)	C55—C56	1.388 (3)
N10—C46	1.376 (3)	C55—H55	0.9500
N10—C50	1.434 (3)	C56—C57	1.379 (3)
N10—C49	1.450 (3)	C56—H56	0.9500
C1—C2	1.494 (3)	C57—C58	1.489 (3)
C1—H1A	0.9800	C58—C59	1.490 (3)
C1—H1B	0.9800	C59—H59A	0.9800
C1—H1C	0.9800	C59—H59B	0.9800
C2—C3	1.489 (3)	C59—H59C	0.9800
C3—C4	1.380 (3)	C60—C61	1.493 (3)
C4—C5	1.387 (3)	C60—H60A	0.9800
C4—H4	0.9500	C60—H60B	0.9800
C5—C6	1.384 (3)	C60—H60C	0.9800
C5—H5	0.9500	C61—C62	1.484 (3)
C6—C7	1.386 (3)	C62—C63	1.384 (3)
C6—H6	0.9500	C63—C64	1.379 (4)
C7—C8	1.486 (3)	C63—H63	0.9500
C8—C9	1.485 (3)	C64—C65	1.382 (4)
C9—H9A	0.9800	C64—H64	0.9500
C9—H9B	0.9800	C65—C66	1.382 (3)
C9—H9C	0.9800	C65—H65	0.9500
C10—C11	1.491 (3)	C66—C67	1.488 (3)
C10—H10A	0.9800	C67—C68	1.489 (3)
C10—H10B	0.9800	C68—H68A	0.9800
C10—H10C	0.9800	C68—H68B	0.9800
C11—C12	1.486 (3)	C68—H68C	0.9800
C12—C13	1.383 (3)	C69—C74	1.384 (3)
C13—C14	1.383 (4)	C69—C70	1.387 (3)
C13—H13	0.9500	C70—C71	1.377 (3)
C14—C15	1.381 (4)	C70—H70	0.9500
C14—H14	0.9500	C71—C72	1.398 (3)
C15—C16	1.383 (3)	C71—H71	0.9500
C15—H15	0.9500	C72—C73	1.400 (3)
C16—C17	1.482 (3)	C73—C74	1.379 (3)
C17—C18	1.488 (3)	C73—H73	0.9500
C18—H18A	0.9800	C74—H74	0.9500
C18—H18B	0.9800	C75—H75A	0.9800
C18—H18C	0.9800	C75—H75B	0.9800
C19—C24	1.377 (3)	C75—H75C	0.9800
C19—C20	1.389 (3)	C76—H76A	0.9800
C20—C21	1.377 (3)	C76—H76B	0.9800
C20—H20	0.9500	C76—H76C	0.9800
C21—C22	1.405 (4)	C77—C78	1.373 (3)
C21—H21	0.9500	C77—C82	1.390 (3)
C22—C23	1.398 (3)	C78—C79	1.383 (3)
C23—C24	1.384 (3)	C78—H78	0.9500
C23—H23	0.9500	C79—C80	1.401 (3)
C24—H24	0.9500	C79—H79	0.9500
C25—H25A	0.9800	C80—C81	1.403 (4)
C25—H25B	0.9800	C81—C82	1.373 (3)
C25—H25C	0.9800	C81—H81	0.9500
C26—H26A	0.9800	C82—H82	0.9500
C26—H26B	0.9800	C83—H83A	0.9800
C26—H26C	0.9800	C83—H83B	0.9800
C27—C32	1.383 (3)	C83—H83C	0.9800
C27—C28	1.387 (3)	C84—H84A	0.9800
C28—C29	1.373 (3)	C84—H84B	0.9800
C28—H28	0.9500	C84—H84C	0.9800
C29—C30	1.402 (3)	C85—C86	1.386 (3)
C29—H29	0.9500	C85—C90	1.387 (3)
C30—C31	1.403 (3)	C86—C87	1.375 (3)
C31—C32	1.376 (3)	C86—H86	0.9500
C31—H31	0.9500	C87—C88	1.404 (3)
C32—H32	0.9500	C87—H87	0.9500
C33—H33A	0.9800	C88—C89	1.405 (3)
C33—H33B	0.9800	C89—C90	1.379 (3)
C33—H33C	0.9800	C89—H89	0.9500
C34—H34A	0.9800	C90—H90	0.9500
C34—H34B	0.9800	C91—H91A	0.9800
C34—H34C	0.9800	C91—H91B	0.9800
C35—C36	1.381 (3)	C91—H91C	0.9800
C35—C40	1.390 (3)	C92—H92A	0.9800
C36—C37	1.379 (3)	C92—H92B	0.9800
C36—H36	0.9500	C92—H92C	0.9800
C37—C38	1.406 (3)	C93—C94	1.379 (3)
C37—H37	0.9500	C93—C98	1.384 (3)
C38—C39	1.404 (3)	C94—C95	1.378 (3)
C39—C40	1.376 (3)	C94—H94	0.9500
C39—H39	0.9500	C95—C96	1.400 (3)
C40—H40	0.9500	C95—H95	0.9500
C41—H41A	0.9800	C96—C97	1.396 (3)
C41—H41B	0.9800	C97—C98	1.377 (3)
C41—H41C	0.9800	C97—H97	0.9500
C42—H42A	0.9800	C98—H98	0.9500
C42—H42B	0.9800	C99—H99A	0.9800
C42—H42C	0.9800	C99—H99B	0.9800
C43—C44	1.378 (3)	C99—H99C	0.9800
C43—C48	1.386 (3)	C100—H10D	0.9800
C44—C45	1.377 (3)	C100—H10E	0.9800
C44—H44	0.9500	C100—H10F	0.9800
C45—C46	1.401 (3)	C1S—Cl5	1.761 (3)
C45—H45	0.9500	C1S—Cl6	1.762 (3)
C46—C47	1.400 (3)	C1S—H1SA	0.9900
C47—C48	1.377 (3)	C1S—H1SB	0.9900
C47—H47	0.9500	C2S—Cl8	1.754 (3)
C48—H48	0.9500	C2S—Cl7	1.804 (3)
C49—H49A	0.9800	C2S—H2SA	0.9900
C49—H49B	0.9800	C2S—H2SB	0.9900
C49—H49C	0.9800	C3S—Cl10	1.747 (3)
C50—H50A	0.9800	C3S—Cl9	1.768 (3)
C50—H50B	0.9800	C3S—H3SA	0.9900
C50—H50C	0.9800	C3S—H3SB	0.9900
Ni2—N12	1.9658 (18)	C4S—Cl12	1.757 (3)
Ni2—N15	1.9711 (18)	C4S—Cl11	1.762 (3)
Ni2—N14	2.0909 (19)	C4S—H4SA	0.9900
Ni2—N11	2.0977 (19)	C4S—H4SB	0.9900
Ni2—N13	2.1188 (19)	O1—H1OA	0.9059
Ni2—N16	2.141 (2)	O1—H1OB	0.8792
N11—C52	1.281 (3)	O2—H2OA	1.0124
N11—C69	1.424 (3)	O2—H2OB	0.9488
N12—C53	1.331 (3)	O3—H3OA	0.8977
N12—C57	1.338 (3)	O3—H3OB	0.8681
N13—C58	1.281 (3)	O4—H4OA	0.9400
N13—C77	1.432 (3)	O4—H4OB	1.0754
			
N2—Ni1—N5	167.57 (8)	N13—Ni2—N16	93.21 (7)
N2—Ni1—N3	77.92 (7)	C52—N11—C69	122.23 (19)
N5—Ni1—N3	111.32 (7)	C52—N11—Ni2	114.90 (15)
N2—Ni1—N4	110.91 (7)	C69—N11—Ni2	122.67 (14)
N5—Ni1—N4	77.83 (8)	C53—N12—C57	121.22 (19)
N3—Ni1—N4	92.14 (7)	C53—N12—Ni2	117.99 (15)
N2—Ni1—N1	76.66 (7)	C57—N12—Ni2	118.85 (15)
N5—Ni1—N1	94.65 (7)	C58—N13—C77	123.3 (2)
N3—Ni1—N1	153.98 (7)	C58—N13—Ni2	115.75 (15)
N4—Ni1—N1	91.75 (7)	C77—N13—Ni2	120.70 (14)
N2—Ni1—N6	94.89 (7)	C61—N14—C85	120.50 (19)
N5—Ni1—N6	76.68 (8)	C61—N14—Ni2	114.47 (15)
N3—Ni1—N6	93.36 (7)	C85—N14—Ni2	125.02 (14)
N4—Ni1—N6	154.19 (7)	C62—N15—C66	121.43 (19)
N1—Ni1—N6	94.25 (7)	C62—N15—Ni2	118.25 (16)
C2—N1—C19	123.0 (2)	C66—N15—Ni2	119.56 (15)
C2—N1—Ni1	115.82 (15)	C67—N16—C93	122.8 (2)
C19—N1—Ni1	120.76 (14)	C67—N16—Ni2	114.91 (16)
C7—N2—C3	121.08 (19)	C93—N16—Ni2	121.82 (14)
C7—N2—Ni1	117.70 (15)	C72—N17—C76	119.4 (2)
C3—N2—Ni1	118.99 (15)	C72—N17—C75	119.1 (2)
C8—N3—C27	121.54 (19)	C76—N17—C75	115.2 (2)
C8—N3—Ni1	115.05 (15)	C80—N18—C83	118.1 (2)
C27—N3—Ni1	123.16 (14)	C80—N18—C84	118.2 (2)
C11—N4—C35	120.56 (19)	C83—N18—C84	117.8 (2)
C11—N4—Ni1	114.91 (16)	C88—N19—C91	118.9 (2)
C35—N4—Ni1	124.53 (14)	C88—N19—C92	118.0 (2)
C12—N5—C16	121.6 (2)	C91—N19—C92	114.2 (2)
C12—N5—Ni1	118.15 (16)	C96—N20—C100	118.0 (2)
C16—N5—Ni1	119.28 (16)	C96—N20—C99	117.7 (2)
C17—N6—C43	123.4 (2)	C100—N20—C99	114.8 (2)
C17—N6—Ni1	115.06 (16)	C52—C51—H51A	109.5
C43—N6—Ni1	121.40 (14)	C52—C51—H51B	109.5
C22—N7—C25	120.1 (2)	H51A—C51—H51B	109.5
C22—N7—C26	120.6 (3)	C52—C51—H51C	109.5
C25—N7—C26	119.2 (2)	H51A—C51—H51C	109.5
C30—N8—C34	120.0 (2)	H51B—C51—H51C	109.5
C30—N8—C33	120.6 (2)	N11—C52—C51	126.4 (2)
C34—N8—C33	118.1 (2)	N11—C52—C53	114.9 (2)
C38—N9—C41	118.4 (2)	C51—C52—C53	118.7 (2)
C38—N9—C42	118.8 (2)	N12—C53—C54	121.0 (2)
C41—N9—C42	114.8 (2)	N12—C53—C52	112.90 (19)
C46—N10—C50	119.4 (2)	C54—C53—C52	126.1 (2)
C46—N10—C49	119.4 (2)	C53—C54—C55	118.4 (2)
C50—N10—C49	118.1 (2)	C53—C54—H54	120.8
C2—C1—H1A	109.5	C55—C54—H54	120.8
C2—C1—H1B	109.5	C54—C55—C56	120.2 (2)
H1A—C1—H1B	109.5	C54—C55—H55	119.9
C2—C1—H1C	109.5	C56—C55—H55	119.9
H1A—C1—H1C	109.5	C57—C56—C55	118.2 (2)
H1B—C1—H1C	109.5	C57—C56—H56	120.9
N1—C2—C3	114.3 (2)	C55—C56—H56	120.9
N1—C2—C1	125.9 (2)	N12—C57—C56	121.0 (2)
C3—C2—C1	119.8 (2)	N12—C57—C58	113.09 (19)
N2—C3—C4	121.0 (2)	C56—C57—C58	125.9 (2)
N2—C3—C2	112.76 (19)	N13—C58—C57	114.1 (2)
C4—C3—C2	126.2 (2)	N13—C58—C59	125.8 (2)
C3—C4—C5	118.1 (2)	C57—C58—C59	120.1 (2)
C3—C4—H4	120.9	C58—C59—H59A	109.5
C5—C4—H4	120.9	C58—C59—H59B	109.5
C6—C5—C4	120.7 (2)	H59A—C59—H59B	109.5
C6—C5—H5	119.7	C58—C59—H59C	109.5
C4—C5—H5	119.7	H59A—C59—H59C	109.5
C5—C6—C7	118.0 (2)	H59B—C59—H59C	109.5
C5—C6—H6	121.0	C61—C60—H60A	109.5
C7—C6—H6	121.0	C61—C60—H60B	109.5
N2—C7—C6	121.0 (2)	H60A—C60—H60B	109.5
N2—C7—C8	112.55 (19)	C61—C60—H60C	109.5
C6—C7—C8	126.4 (2)	H60A—C60—H60C	109.5
N3—C8—C9	126.2 (2)	H60B—C60—H60C	109.5
N3—C8—C7	115.0 (2)	N14—C61—C62	115.5 (2)
C9—C8—C7	118.7 (2)	N14—C61—C60	126.2 (2)
C8—C9—H9A	109.5	C62—C61—C60	118.2 (2)
C8—C9—H9B	109.5	N15—C62—C63	120.7 (2)
H9A—C9—H9B	109.5	N15—C62—C61	113.01 (19)
C8—C9—H9C	109.5	C63—C62—C61	126.2 (2)
H9A—C9—H9C	109.5	C64—C63—C62	118.3 (2)
H9B—C9—H9C	109.5	C64—C63—H63	120.9
C11—C10—H10A	109.5	C62—C63—H63	120.9
C11—C10—H10B	109.5	C63—C64—C65	120.5 (2)
H10A—C10—H10B	109.5	C63—C64—H64	119.8
C11—C10—H10C	109.5	C65—C64—H64	119.8
H10A—C10—H10C	109.5	C64—C65—C66	118.3 (2)
H10B—C10—H10C	109.5	C64—C65—H65	120.9
N4—C11—C12	114.9 (2)	C66—C65—H65	120.9
N4—C11—C10	126.9 (2)	N15—C66—C65	120.7 (2)
C12—C11—C10	118.1 (2)	N15—C66—C67	113.43 (19)
N5—C12—C13	120.5 (2)	C65—C66—C67	125.8 (2)
N5—C12—C11	113.50 (19)	N16—C67—C66	114.8 (2)
C13—C12—C11	126.0 (2)	N16—C67—C68	126.2 (2)
C12—C13—C14	118.7 (2)	C66—C67—C68	118.9 (2)
C12—C13—H13	120.7	C67—C68—H68A	109.5
C14—C13—H13	120.7	C67—C68—H68B	109.5
C15—C14—C13	120.0 (2)	H68A—C68—H68B	109.5
C15—C14—H14	120.0	C67—C68—H68C	109.5
C13—C14—H14	120.0	H68A—C68—H68C	109.5
C14—C15—C16	118.5 (2)	H68B—C68—H68C	109.5
C14—C15—H15	120.7	C74—C69—C70	118.5 (2)
C16—C15—H15	120.7	C74—C69—N11	119.0 (2)
N5—C16—C15	120.6 (2)	C70—C69—N11	122.3 (2)
N5—C16—C17	113.43 (19)	C71—C70—C69	120.9 (2)
C15—C16—C17	126.0 (2)	C71—C70—H70	119.6
N6—C17—C16	114.8 (2)	C69—C70—H70	119.6
N6—C17—C18	126.1 (2)	C70—C71—C72	121.1 (2)
C16—C17—C18	119.1 (2)	C70—C71—H71	119.4
C17—C18—H18A	109.5	C72—C71—H71	119.4
C17—C18—H18B	109.5	N17—C72—C71	121.9 (2)
H18A—C18—H18B	109.5	N17—C72—C73	120.5 (2)
C17—C18—H18C	109.5	C71—C72—C73	117.6 (2)
H18A—C18—H18C	109.5	C74—C73—C72	120.8 (2)
H18B—C18—H18C	109.5	C74—C73—H73	119.6
C24—C19—C20	119.3 (2)	C72—C73—H73	119.6
C24—C19—N1	119.3 (2)	C73—C74—C69	121.1 (2)
C20—C19—N1	120.5 (2)	C73—C74—H74	119.4
C21—C20—C19	120.5 (2)	C69—C74—H74	119.4
C21—C20—H20	119.7	N17—C75—H75A	109.5
C19—C20—H20	119.7	N17—C75—H75B	109.5
C20—C21—C22	120.8 (2)	H75A—C75—H75B	109.5
C20—C21—H21	119.6	N17—C75—H75C	109.5
C22—C21—H21	119.6	H75A—C75—H75C	109.5
N7—C22—C23	120.7 (2)	H75B—C75—H75C	109.5
N7—C22—C21	121.4 (2)	N17—C76—H76A	109.5
C23—C22—C21	117.9 (2)	N17—C76—H76B	109.5
C24—C23—C22	120.7 (2)	H76A—C76—H76B	109.5
C24—C23—H23	119.6	N17—C76—H76C	109.5
C22—C23—H23	119.6	H76A—C76—H76C	109.5
C19—C24—C23	120.7 (2)	H76B—C76—H76C	109.5
C19—C24—H24	119.6	C78—C77—C82	119.0 (2)
C23—C24—H24	119.6	C78—C77—N13	119.4 (2)
N7—C25—H25A	109.5	C82—C77—N13	120.6 (2)
N7—C25—H25B	109.5	C77—C78—C79	121.0 (2)
H25A—C25—H25B	109.5	C77—C78—H78	119.5
N7—C25—H25C	109.5	C79—C78—H78	119.5
H25A—C25—H25C	109.5	C78—C79—C80	120.8 (2)
H25B—C25—H25C	109.5	C78—C79—H79	119.6
N7—C26—H26A	109.5	C80—C79—H79	119.6
N7—C26—H26B	109.5	N18—C80—C79	120.0 (2)
H26A—C26—H26B	109.5	N18—C80—C81	122.6 (2)
N7—C26—H26C	109.5	C79—C80—C81	117.4 (2)
H26A—C26—H26C	109.5	C82—C81—C80	121.1 (2)
H26B—C26—H26C	109.5	C82—C81—H81	119.4
C32—C27—C28	118.5 (2)	C80—C81—H81	119.4
C32—C27—N3	119.5 (2)	C81—C82—C77	120.7 (2)
C28—C27—N3	121.9 (2)	C81—C82—H82	119.7
C29—C28—C27	121.3 (2)	C77—C82—H82	119.7
C29—C28—H28	119.4	N18—C83—H83A	109.5
C27—C28—H28	119.4	N18—C83—H83B	109.5
C28—C29—C30	120.7 (2)	H83A—C83—H83B	109.5
C28—C29—H29	119.6	N18—C83—H83C	109.5
C30—C29—H29	119.6	H83A—C83—H83C	109.5
N8—C30—C29	121.5 (2)	H83B—C83—H83C	109.5
N8—C30—C31	121.0 (2)	N18—C84—H84A	109.5
C29—C30—C31	117.5 (2)	N18—C84—H84B	109.5
C32—C31—C30	121.1 (2)	H84A—C84—H84B	109.5
C32—C31—H31	119.5	N18—C84—H84C	109.5
C30—C31—H31	119.5	H84A—C84—H84C	109.5
C31—C32—C27	120.8 (2)	H84B—C84—H84C	109.5
C31—C32—H32	119.6	C86—C85—C90	118.7 (2)
C27—C32—H32	119.6	C86—C85—N14	121.3 (2)
N8—C33—H33A	109.5	C90—C85—N14	120.0 (2)
N8—C33—H33B	109.5	C87—C86—C85	121.0 (2)
H33A—C33—H33B	109.5	C87—C86—H86	119.5
N8—C33—H33C	109.5	C85—C86—H86	119.5
H33A—C33—H33C	109.5	C86—C87—C88	121.2 (2)
H33B—C33—H33C	109.5	C86—C87—H87	119.4
N8—C34—H34A	109.5	C88—C87—H87	119.4
N8—C34—H34B	109.5	N19—C88—C87	121.1 (2)
H34A—C34—H34B	109.5	N19—C88—C89	121.8 (2)
N8—C34—H34C	109.5	C87—C88—C89	117.1 (2)
H34A—C34—H34C	109.5	C90—C89—C88	121.2 (2)
H34B—C34—H34C	109.5	C90—C89—H89	119.4
C36—C35—C40	118.9 (2)	C88—C89—H89	119.4
C36—C35—N4	120.1 (2)	C89—C90—C85	120.8 (2)
C40—C35—N4	121.0 (2)	C89—C90—H90	119.6
C37—C36—C35	120.9 (2)	C85—C90—H90	119.6
C37—C36—H36	119.5	N19—C91—H91A	109.5
C35—C36—H36	119.5	N19—C91—H91B	109.5
C36—C37—C38	121.2 (2)	H91A—C91—H91B	109.5
C36—C37—H37	119.4	N19—C91—H91C	109.5
C38—C37—H37	119.4	H91A—C91—H91C	109.5
N9—C38—C39	121.3 (2)	H91B—C91—H91C	109.5
N9—C38—C37	121.7 (2)	N19—C92—H92A	109.5
C39—C38—C37	116.9 (2)	N19—C92—H92B	109.5
C40—C39—C38	121.6 (2)	H92A—C92—H92B	109.5
C40—C39—H39	119.2	N19—C92—H92C	109.5
C38—C39—H39	119.2	H92A—C92—H92C	109.5
C39—C40—C35	120.5 (2)	H92B—C92—H92C	109.5
C39—C40—H40	119.7	C94—C93—C98	118.3 (2)
C35—C40—H40	119.7	C94—C93—N16	121.3 (2)
N9—C41—H41A	109.5	C98—C93—N16	119.8 (2)
N9—C41—H41B	109.5	C95—C94—C93	120.9 (2)
H41A—C41—H41B	109.5	C95—C94—H94	119.5
N9—C41—H41C	109.5	C93—C94—H94	119.5
H41A—C41—H41C	109.5	C94—C95—C96	121.7 (2)
H41B—C41—H41C	109.5	C94—C95—H95	119.2
N9—C42—H42A	109.5	C96—C95—H95	119.2
N9—C42—H42B	109.5	N20—C96—C97	122.2 (2)
H42A—C42—H42B	109.5	N20—C96—C95	121.4 (2)
N9—C42—H42C	109.5	C97—C96—C95	116.4 (2)
H42A—C42—H42C	109.5	C98—C97—C96	121.7 (2)
H42B—C42—H42C	109.5	C98—C97—H97	119.2
C44—C43—C48	118.5 (2)	C96—C97—H97	119.2
C44—C43—N6	121.2 (2)	C97—C98—C93	120.9 (2)
C48—C43—N6	119.5 (2)	C97—C98—H98	119.5
C45—C44—C43	121.0 (2)	C93—C98—H98	119.5
C45—C44—H44	119.5	N20—C99—H99A	109.5
C43—C44—H44	119.5	N20—C99—H99B	109.5
C44—C45—C46	121.4 (2)	H99A—C99—H99B	109.5
C44—C45—H45	119.3	N20—C99—H99C	109.5
C46—C45—H45	119.3	H99A—C99—H99C	109.5
N10—C46—C47	121.7 (2)	H99B—C99—H99C	109.5
N10—C46—C45	121.5 (2)	N20—C100—H10D	109.5
C47—C46—C45	116.8 (2)	N20—C100—H10E	109.5
C48—C47—C46	121.3 (2)	H10D—C100—H10E	109.5
C48—C47—H47	119.4	N20—C100—H10F	109.5
C46—C47—H47	119.4	H10D—C100—H10F	109.5
C47—C48—C43	120.9 (2)	H10E—C100—H10F	109.5
C47—C48—H48	119.5	Cl5—C1S—Cl6	110.65 (16)
C43—C48—H48	119.5	Cl5—C1S—H1SA	109.5
N10—C49—H49A	109.5	Cl6—C1S—H1SA	109.5
N10—C49—H49B	109.5	Cl5—C1S—H1SB	109.5
H49A—C49—H49B	109.5	Cl6—C1S—H1SB	109.5
N10—C49—H49C	109.5	H1SA—C1S—H1SB	108.1
H49A—C49—H49C	109.5	Cl8—C2S—Cl7	109.50 (16)
H49B—C49—H49C	109.5	Cl8—C2S—H2SA	109.8
N10—C50—H50A	109.5	Cl7—C2S—H2SA	109.8
N10—C50—H50B	109.5	Cl8—C2S—H2SB	109.8
H50A—C50—H50B	109.5	Cl7—C2S—H2SB	109.8
N10—C50—H50C	109.5	H2SA—C2S—H2SB	108.2
H50A—C50—H50C	109.5	Cl10—C3S—Cl9	112.50 (15)
H50B—C50—H50C	109.5	Cl10—C3S—H3SA	109.1
N12—Ni2—N15	167.94 (8)	Cl9—C3S—H3SA	109.1
N12—Ni2—N14	110.67 (7)	Cl10—C3S—H3SB	109.1
N15—Ni2—N14	78.01 (7)	Cl9—C3S—H3SB	109.1
N12—Ni2—N11	77.79 (7)	H3SA—C3S—H3SB	107.8
N15—Ni2—N11	111.03 (7)	Cl12—C4S—Cl11	110.70 (16)
N14—Ni2—N11	91.70 (7)	Cl12—C4S—H4SA	109.5
N12—Ni2—N13	76.89 (7)	Cl11—C4S—H4SA	109.5
N15—Ni2—N13	94.86 (7)	Cl12—C4S—H4SB	109.5
N14—Ni2—N13	91.88 (7)	Cl11—C4S—H4SB	109.5
N11—Ni2—N13	154.04 (7)	H4SA—C4S—H4SB	108.1
N12—Ni2—N16	94.74 (7)	H1OA—O1—H1OB	101.9
N15—Ni2—N16	76.75 (8)	H2OA—O2—H2OB	106.9
N14—Ni2—N16	154.58 (7)	H3OA—O3—H3OB	107.3
N11—Ni2—N16	94.50 (7)	H4OA—O4—H4OB	97.4
			
C19—N1—C2—C3	−174.7 (2)	C69—N11—C52—C51	8.0 (4)
Ni1—N1—C2—C3	−1.7 (3)	Ni2—N11—C52—C51	−177.1 (2)
C19—N1—C2—C1	6.6 (4)	C69—N11—C52—C53	−174.92 (19)
Ni1—N1—C2—C1	179.59 (18)	Ni2—N11—C52—C53	0.0 (3)
C7—N2—C3—C4	0.9 (3)	C57—N12—C53—C54	0.7 (3)
Ni1—N2—C3—C4	163.54 (17)	Ni2—N12—C53—C54	164.72 (18)
C7—N2—C3—C2	−176.91 (19)	C57—N12—C53—C52	−178.1 (2)
Ni1—N2—C3—C2	−14.3 (2)	Ni2—N12—C53—C52	−14.1 (2)
N1—C2—C3—N2	9.9 (3)	N11—C52—C53—N12	8.8 (3)
C1—C2—C3—N2	−171.3 (2)	C51—C52—C53—N12	−173.9 (2)
N1—C2—C3—C4	−167.8 (2)	N11—C52—C53—C54	−170.0 (2)
C1—C2—C3—C4	11.0 (4)	C51—C52—C53—C54	7.4 (4)
N2—C3—C4—C5	−1.4 (3)	N12—C53—C54—C55	0.2 (4)
C2—C3—C4—C5	176.1 (2)	C52—C53—C54—C55	178.8 (2)
C3—C4—C5—C6	1.0 (4)	C53—C54—C55—C56	−0.2 (4)
C4—C5—C6—C7	−0.1 (4)	C54—C55—C56—C57	−0.6 (4)
C3—N2—C7—C6	0.0 (3)	C53—N12—C57—C56	−1.6 (3)
Ni1—N2—C7—C6	−162.83 (17)	Ni2—N12—C57—C56	−165.45 (17)
C3—N2—C7—C8	177.9 (2)	C53—N12—C57—C58	177.1 (2)
Ni1—N2—C7—C8	15.1 (2)	Ni2—N12—C57—C58	13.2 (3)
C5—C6—C7—N2	−0.4 (3)	C55—C56—C57—N12	1.5 (3)
C5—C6—C7—C8	−178.0 (2)	C55—C56—C57—C58	−177.0 (2)
C27—N3—C8—C9	−8.4 (4)	C77—N13—C58—C57	173.8 (2)
Ni1—N3—C8—C9	177.17 (19)	Ni2—N13—C58—C57	−0.4 (3)
C27—N3—C8—C7	173.92 (19)	C77—N13—C58—C59	−7.6 (4)
Ni1—N3—C8—C7	−0.5 (3)	Ni2—N13—C58—C59	178.22 (18)
N2—C7—C8—N3	−9.1 (3)	N12—C57—C58—N13	−7.8 (3)
C6—C7—C8—N3	168.6 (2)	C56—C57—C58—N13	170.8 (2)
N2—C7—C8—C9	173.1 (2)	N12—C57—C58—C59	173.4 (2)
C6—C7—C8—C9	−9.2 (4)	C56—C57—C58—C59	−8.0 (4)
C35—N4—C11—C12	173.98 (19)	C85—N14—C61—C62	−173.63 (19)
Ni1—N4—C11—C12	−6.3 (2)	Ni2—N14—C61—C62	7.3 (2)
C35—N4—C11—C10	−10.6 (3)	C85—N14—C61—C60	11.3 (3)
Ni1—N4—C11—C10	169.21 (19)	Ni2—N14—C61—C60	−167.74 (19)
C16—N5—C12—C13	−3.5 (3)	C66—N15—C62—C63	3.5 (3)
Ni1—N5—C12—C13	−172.13 (17)	Ni2—N15—C62—C63	173.47 (17)
C16—N5—C12—C11	175.21 (19)	C66—N15—C62—C61	−175.69 (19)
Ni1—N5—C12—C11	6.6 (3)	Ni2—N15—C62—C61	−5.7 (3)
N4—C11—C12—N5	0.2 (3)	N14—C61—C62—N15	−1.5 (3)
C10—C11—C12—N5	−175.7 (2)	C60—C61—C62—N15	174.0 (2)
N4—C11—C12—C13	178.8 (2)	N14—C61—C62—C63	179.4 (2)
C10—C11—C12—C13	2.9 (3)	C60—C61—C62—C63	−5.1 (3)
N5—C12—C13—C14	1.1 (4)	N15—C62—C63—C64	−0.3 (4)
C11—C12—C13—C14	−177.5 (2)	C61—C62—C63—C64	178.7 (2)
C12—C13—C14—C15	1.7 (4)	C62—C63—C64—C65	−2.1 (4)
C13—C14—C15—C16	−2.0 (4)	C63—C64—C65—C66	1.6 (4)
C12—N5—C16—C15	3.1 (3)	C62—N15—C66—C65	−4.0 (3)
Ni1—N5—C16—C15	171.65 (17)	Ni2—N15—C66—C65	−173.90 (17)
C12—N5—C16—C17	−175.3 (2)	C62—N15—C66—C67	173.76 (19)
Ni1—N5—C16—C17	−6.8 (3)	Ni2—N15—C66—C67	3.9 (3)
C14—C15—C16—N5	−0.3 (3)	C64—C65—C66—N15	1.4 (3)
C14—C15—C16—C17	177.9 (2)	C64—C65—C66—C67	−176.0 (2)
C43—N6—C17—C16	−178.06 (19)	C93—N16—C67—C66	−178.7 (2)
Ni1—N6—C17—C16	6.1 (2)	Ni2—N16—C67—C66	−6.6 (2)
C43—N6—C17—C18	4.2 (4)	C93—N16—C67—C68	−1.7 (4)
Ni1—N6—C17—C18	−171.64 (19)	Ni2—N16—C67—C68	170.43 (18)
N5—C16—C17—N6	0.0 (3)	N15—C66—C67—N16	2.2 (3)
C15—C16—C17—N6	−178.4 (2)	C65—C66—C67—N16	179.8 (2)
N5—C16—C17—C18	177.9 (2)	N15—C66—C67—C68	−175.1 (2)
C15—C16—C17—C18	−0.5 (4)	C65—C66—C67—C68	2.6 (3)
C2—N1—C19—C24	84.7 (3)	C52—N11—C69—C74	−130.9 (2)
Ni1—N1—C19—C24	−87.9 (2)	Ni2—N11—C69—C74	54.5 (3)
C2—N1—C19—C20	−106.2 (3)	C52—N11—C69—C70	54.3 (3)
Ni1—N1—C19—C20	81.2 (2)	Ni2—N11—C69—C70	−120.3 (2)
C24—C19—C20—C21	0.2 (3)	C74—C69—C70—C71	0.9 (3)
N1—C19—C20—C21	−168.9 (2)	N11—C69—C70—C71	175.7 (2)
C19—C20—C21—C22	0.6 (4)	C69—C70—C71—C72	0.1 (4)
C25—N7—C22—C23	2.9 (4)	C76—N17—C72—C71	−168.5 (2)
C26—N7—C22—C23	−173.3 (3)	C75—N17—C72—C71	−17.6 (3)
C25—N7—C22—C21	−176.1 (3)	C76—N17—C72—C73	13.7 (3)
C26—N7—C22—C21	7.6 (4)	C75—N17—C72—C73	164.6 (2)
C20—C21—C22—N7	177.5 (3)	C70—C71—C72—N17	−178.1 (2)
C20—C21—C22—C23	−1.5 (4)	C70—C71—C72—C73	−0.3 (3)
N7—C22—C23—C24	−177.4 (2)	N17—C72—C73—C74	177.4 (2)
C21—C22—C23—C24	1.7 (4)	C71—C72—C73—C74	−0.4 (3)
C20—C19—C24—C23	−0.1 (3)	C72—C73—C74—C69	1.4 (4)
N1—C19—C24—C23	169.1 (2)	C70—C69—C74—C73	−1.7 (3)
C22—C23—C24—C19	−0.9 (4)	N11—C69—C74—C73	−176.7 (2)
C8—N3—C27—C32	130.6 (2)	C58—N13—C77—C78	−86.4 (3)
Ni1—N3—C27—C32	−55.5 (3)	Ni2—N13—C77—C78	87.6 (2)
C8—N3—C27—C28	−53.9 (3)	C58—N13—C77—C82	105.1 (3)
Ni1—N3—C27—C28	120.1 (2)	Ni2—N13—C77—C82	−81.0 (2)
C32—C27—C28—C29	−1.0 (3)	C82—C77—C78—C79	0.6 (3)
N3—C27—C28—C29	−176.6 (2)	N13—C77—C78—C79	−168.2 (2)
C27—C28—C29—C30	−0.9 (4)	C77—C78—C79—C80	0.2 (4)
C34—N8—C30—C29	−174.7 (2)	C83—N18—C80—C79	−25.9 (4)
C33—N8—C30—C29	−8.3 (4)	C84—N18—C80—C79	−178.5 (3)
C34—N8—C30—C31	4.8 (4)	C83—N18—C80—C81	154.1 (3)
C33—N8—C30—C31	171.2 (2)	C84—N18—C80—C81	1.4 (4)
C28—C29—C30—N8	−178.9 (2)	C78—C79—C80—N18	178.9 (2)
C28—C29—C30—C31	1.6 (3)	C78—C79—C80—C81	−1.0 (4)
N8—C30—C31—C32	−179.9 (2)	N18—C80—C81—C82	−178.7 (2)
C29—C30—C31—C32	−0.4 (4)	C79—C80—C81—C82	1.2 (4)
C30—C31—C32—C27	−1.5 (4)	C80—C81—C82—C77	−0.5 (4)
C28—C27—C32—C31	2.2 (3)	C78—C77—C82—C81	−0.4 (3)
N3—C27—C32—C31	177.9 (2)	N13—C77—C82—C81	168.2 (2)
C11—N4—C35—C36	129.5 (2)	C61—N14—C85—C86	53.4 (3)
Ni1—N4—C35—C36	−50.3 (3)	Ni2—N14—C85—C86	−127.7 (2)
C11—N4—C35—C40	−53.0 (3)	C61—N14—C85—C90	−128.8 (2)
Ni1—N4—C35—C40	127.2 (2)	Ni2—N14—C85—C90	50.1 (3)
C40—C35—C36—C37	2.4 (3)	C90—C85—C86—C87	1.8 (4)
N4—C35—C36—C37	180.0 (2)	N14—C85—C86—C87	179.5 (2)
C35—C36—C37—C38	−2.2 (4)	C85—C86—C87—C88	−0.6 (4)
C41—N9—C38—C39	170.1 (2)	C91—N19—C88—C87	−19.4 (3)
C42—N9—C38—C39	23.0 (3)	C92—N19—C88—C87	−164.9 (2)
C41—N9—C38—C37	−13.3 (3)	C91—N19—C88—C89	162.7 (2)
C42—N9—C38—C37	−160.4 (2)	C92—N19—C88—C89	17.2 (3)
C36—C37—C38—N9	−175.8 (2)	C86—C87—C88—N19	−177.7 (2)
C36—C37—C38—C39	0.9 (3)	C86—C87—C88—C89	0.3 (3)
N9—C38—C39—C40	176.7 (2)	N19—C88—C89—C90	176.7 (2)
C37—C38—C39—C40	0.0 (3)	C87—C88—C89—C90	−1.2 (3)
C38—C39—C40—C35	0.3 (4)	C88—C89—C90—C85	2.5 (4)
C36—C35—C40—C39	−1.5 (3)	C86—C85—C90—C89	−2.7 (3)
N4—C35—C40—C39	−179.0 (2)	N14—C85—C90—C89	179.5 (2)
C17—N6—C43—C44	−89.2 (3)	C67—N16—C93—C94	81.9 (3)
Ni1—N6—C43—C44	86.4 (2)	Ni2—N16—C93—C94	−89.7 (2)
C17—N6—C43—C48	101.2 (3)	C67—N16—C93—C98	−107.3 (3)
Ni1—N6—C43—C48	−83.2 (2)	Ni2—N16—C93—C98	81.2 (2)
C48—C43—C44—C45	1.2 (4)	C98—C93—C94—C95	−1.0 (4)
N6—C43—C44—C45	−168.5 (2)	N16—C93—C94—C95	170.0 (2)
C43—C44—C45—C46	−0.4 (4)	C93—C94—C95—C96	−0.5 (4)
C50—N10—C46—C47	8.6 (4)	C100—N20—C96—C97	−7.7 (4)
C49—N10—C46—C47	168.5 (2)	C99—N20—C96—C97	−152.2 (2)
C50—N10—C46—C45	−172.0 (3)	C100—N20—C96—C95	174.5 (2)
C49—N10—C46—C45	−12.1 (4)	C99—N20—C96—C95	30.0 (3)
C44—C45—C46—N10	179.5 (2)	C94—C95—C96—N20	−179.9 (2)
C44—C45—C46—C47	−1.1 (4)	C94—C95—C96—C97	2.2 (4)
N10—C46—C47—C48	−178.8 (2)	N20—C96—C97—C98	179.6 (2)
C45—C46—C47—C48	1.8 (4)	C95—C96—C97—C98	−2.5 (4)
C46—C47—C48—C43	−1.0 (4)	C96—C97—C98—C93	1.2 (4)
C44—C43—C48—C47	−0.6 (4)	C94—C93—C98—C97	0.7 (4)
N6—C43—C48—C47	169.4 (2)	N16—C93—C98—C97	−170.5 (2)

### Hydrogen-bond geometry (Å, º)

**Table:**

*D*—H···*A*	*D*—H	H···*A*	*D*···*A*	*D*—H···*A*
O1—H1*OA*···Cl1	0.91	2.33	3.2381 (19)	177
O1—H1*OB*···Cl2	0.88	2.31	3.188 (2)	177
O2—H2*OA*···Cl1	1.01	2.18	3.192 (2)	175
O2—H2*OB*···Cl2	0.95	2.32	3.2691 (19)	177
O3—H3*OA*···Cl4	0.90	2.30	3.198 (2)	178
O3—H3*OB*···Cl3	0.87	2.34	3.201 (2)	174
O4—H4*OB*···Cl3	1.08	2.12	3.180 (2)	168
O4—H4*OA*···Cl4	0.94	2.25	3.192 (2)	177
C1*S*—H1*SB*···Cl3	0.99	2.47	3.422 (3)	162
C2*S*—H2*SA*···Cl4	0.99	2.81	3.743 (4)	158
C2*S*—H2*SB*···Cl1^i^	0.99	2.66	3.642 (3)	169
C3*S*—H3*SA*···N17^ii^	0.99	2.42	3.403 (4)	173
C3*S*—H3*SB*···Cl2	0.99	2.49	3.447 (3)	163
C4*S*—H4*SA*···Cl4	0.99	2.54	3.527 (3)	176

Symmetry codes: (i) *x*, *y*+1, *z*; (ii) *x*−1, *y*, *z*.
